# FAM120A deficiency improves resistance to cisplatin in gastric cancer by promoting ferroptosis

**DOI:** 10.1038/s42003-024-06097-6

**Published:** 2024-04-02

**Authors:** Liangbo Niu, Yi Li, Guixiang Huang, Wei Huang, Jing Fu, Lu Feng

**Affiliations:** 1 Department of Emergency surgery, Sichuan Provincial People’s Hospital, University of Electronic Science and Technology of China, Chengdu, 610072 Sichuan China; 2 Department of Emergency Medicine, Sichuan Provincial People’s Hospital, University of Electronic Science and Technology of China, Chengdu, 610072 Sichuan China; 3 Department of Geriatric Medicine and Gastroenterology, Sichuan Provincial People’s Hospital, University of Electronic Science and Technology of China, Chengdu, 610072 Sichuan China

**Keywords:** Biomarkers, Gastric cancer, Cell signalling

## Abstract

The occurrence of chemoresistance is an inescapable obstacle affecting the clinical efficacy of cisplatin in gastric cancer (GC). Exploring the regulatory mechanism of cisplatin resistance will help to provide potential effective targets for improving the prognosis of gastric cancer patients. Here, we find that FAM120A is upregulated in GC tissues and higher in cisplatin-resistant GC tissues, and its high expression is positively correlated with the poor outcome of GC patients. Functional studies indicate that FAM120A confers chemoresistance to GC cells by inhibiting ferroptosis. Mechanically, METTL3-induced m6A modification and YTHDC1-induced stability of *FAM120A* mRNA enhance FAM120A expression. FAM120A inhibits ferroptosis by binding *SLC7A11* mRNA and enhancing its stability. FAM120A deficiency enhances cisplatin sensitivity by promoting ferroptosis in vivo. These results reveal the function of FAM120A in chemotherapy tolerance and targeting FAM120A is an effective strategy to alleviate cisplatin resistance in GC.

## Abstract (web summary)

N6-methyladenosine modification of FAM120A enhanced cisplatin resistance by inhibiting ferroptosis and targeting FAM120A is an effective strategy to alleviate cisplatin resistance in gastric cancer.

## Introduction

Gastric cancer (GC) is one of the most commonly diagnosed tumors and the fourth leading cause of cancer-related deaths worldwide^1^. Despite the rapid development of diagnostic and treatment strategies for GC, the prognosis of patients with GC is still unsatisfactory^2^. Due to the advanced stage at the time of initial diagnosis and poor response to treatment, most patients with GC have a poor prognosis^3^. Cisplatin-based chemotherapy remains the mainstream treatment for advanced GC^4,5^, but the emergence of chemoresistance severely limits its efficacy. Therefore, exploring the regulatory mechanism of cisplatin resistance and enhancing the sensitivity of GC to cisplatin will help to improve the prognosis of GC patients.

Insensitivity to cell death stimulus is one of the key characteristics of tumors and one of the main causes of chemotherapy resistance in GC^6,7^. Ferroptosis is a recently discovered mechanism of cell death, characterized by lipid peroxidation and GSH depletion mediated by iron metabolism^8,9^. Ferroptosis primarily arises from the deactivation of the cellular antioxidant mechanism, particularly the SCL7A11 (solute carrier family 7 member 11)-GSH (glutathione)-GPX4 (glutathione peroxidase 4)-dependent defense system against oxidative stress, increasing lipid hydroperoxide buildup and ferroptosis^10^. The import of extracellular cystine across the cell membrane is facilitated by the system SCL7A11 antiporter, leading to its conversion into intracellular cysteine. Cellular internalization of cystine results in its swift conversion to cysteine, which serves as a precursor with limited availability for the synthesis of GSH. Consequently, reduced GSH is utilized by GPX4 as a co-factor to diminish lipid hydroperoxides into lipid alcohols, thereby safeguarding cells against ferroptosis induced by lipid peroxidation. A growing body of recent research has demonstrated that enhancing intracellular Fe2+ levels, ROS, depleting antioxidant GSH, or inactivating GPX4 in GC cells can potentially enhance the efficacy of clinical interventions for GC^11,12^. It has been reported that ferroptosis is increased by cisplatin treatment and that a ferroptosis inhibitor partially rescues the cell death of GC cells induced by cisplatin^13^. Zhang et al. indicated that miR-522 from cancer-associated fibroblasts inhibits ferroptosis and helps chemo-resistance in GC^14^. In addition, activating transcription factor 3 (ATF3) overexpression alleviates cisplatin resistance in GC by promoting ferroptosis^15^. Thus, targeting ferroptosis could be an effective strategy to inhibit tumor development and improve chemotherapy tolerance in GC patients.

*FAM120A* (also named OSSA/C9ORF10) is mapped to chromosome 9q22.31, which was first reported to be a component of Purα-containing mRNP complexes^16^. The association between *FAM120A* and GC has been reported by Tanaka et al.^17^. They find that FAM120A is abundantly expressed in the cytoplasm of the GC cells and elevated FAM120A is observed in the scirrhous-type GC tissues compared with normal gastric mucosa. Mechanically, FAM120A guards GC cells from oxidative stress-induced apoptosis by activation of Src family kinases (SFKs)/PI3-kinase-Akt pathway. On the other hand, they find that the carboxyl terminus of FAM120A protein directly binds to *IGF-II* mRNA in a tyrosine phosphorylation-independent manner in GC. The overexpression of FAM120A significantly increases IGF-II protein in the culture medium in gastric cancer cells. Moreover, they display that suppression of FAM120A expression inhibits the growth and invasion of GC in nude mice. However, the association between FAM120A and chemotherapy tolerance in GC has not been established.

In this study, we identified that FAM120A was upregulated in GC tissues and was higher in cisplatin-resistant patients. *FAM120A* expression was controlled by METTL3-induced m6A modification and YTHDC1-mediated mRNA stabilization. We found that FAM120A inhibited ferroptosis and promoted cisplatin resistance in GC cells by enhancing the stability of *SCL7A11* mRNA. FAM120A deficiency enhanced cisplatin sensitivity by promoting ferroptosis in vivo. Here, we demonstrate that FAM120A depletion may be a prospective strategy for improving the chemoresistance of cisplatin in GC patients.

## Results

### FAM120A is increased and drives GC growth

**Fig. 1:**
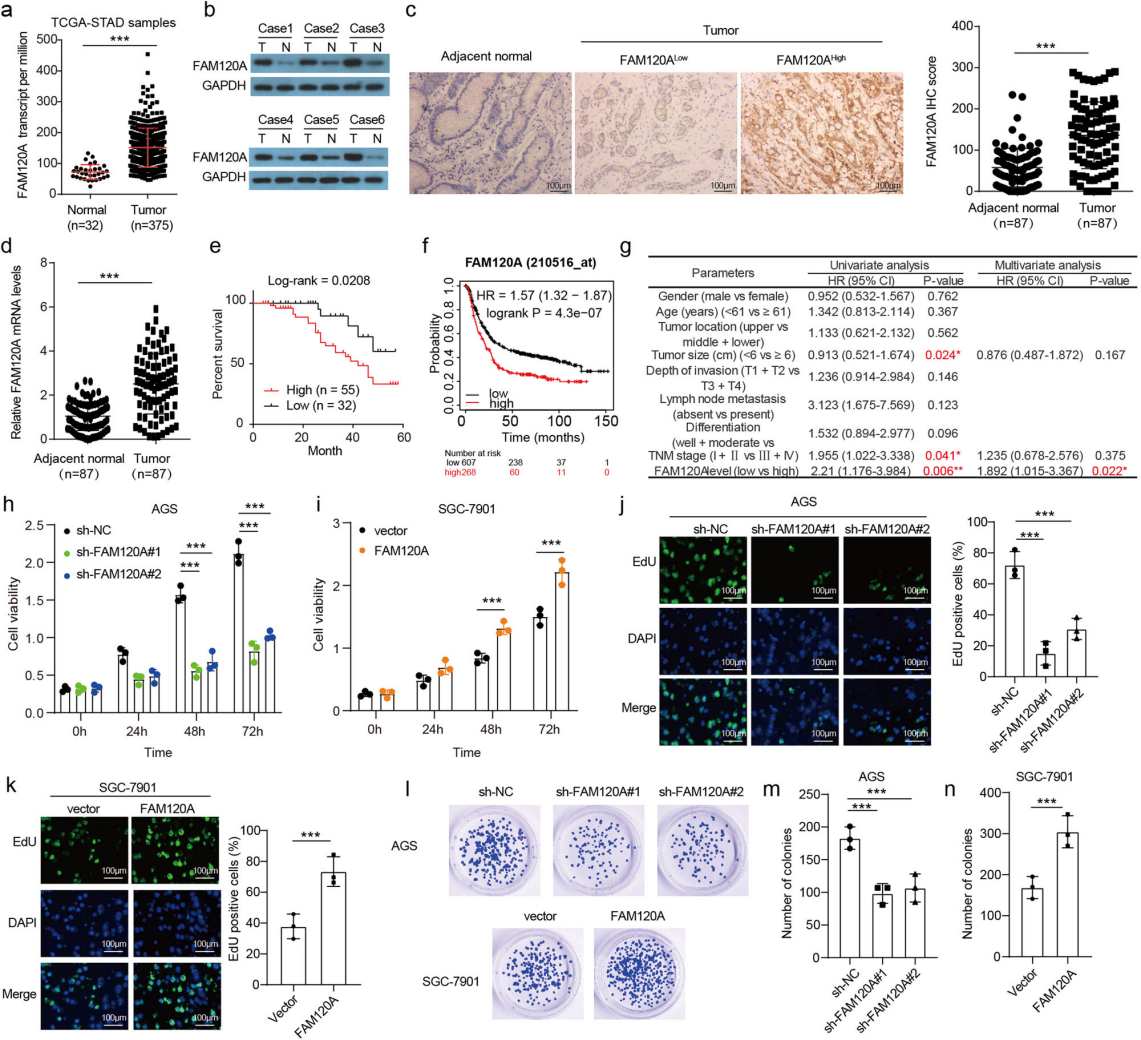
FAM120A was upregulated and promoted GC growth in vitro. **a** FAM120A levels were evaluated based on publicly available TCGA-STAD databases. **b** FAM120A levels in in-house 6 pairs fresh GC and adjacent normal tissues were detected by Western blotting. **c** FAM120A levels in in-house GC and adjacent normal tissues were detected by IHC. Scale bars = 100 μm. **d** FAM120A levels in in-house GC and adjacent normal tissues were measured by qRT‒PCR. **e** Overall survival curve of GC patients with low and high FAM120A levels in the in-house cohort. **f** Kaplan‒Meier survival curve of FAM120A level was obtained from the publicly available KM plotter database. **g** Univariate analysis and multivariate analysis to evaluate the risk factors for GC prognosis in the in-house cohort. **h**, **i** The effect of FAM120A overexpression or depletion on GC cell viability was assessed by CCK-8 (*N* = 3). **j**, **k** The effect of FAM120A overexpression or depletion on GC cell proliferation was assessed by EdU staining (*N* = 3). Scale bars = 100 μm. **l**–**n** The effect of FAM120A overexpression or depletion on GC cell colony formation was assessed by clone formation assay (*N* = 3). Statistical significance was determined using Student’s *t*-test (**a**, **c**, **d**, **i**, **k**, **n**), the log-rank test (**e**, **f**), one-way ANOVA followed by Tukey multiple comparisons (**h**, **j**, **m**). ***p < 0.001.

To uncover the function of *FAM120A* in GC, we first evaluated *FAM120A* levels by using the TCGA database and discovered an increase in *FAM120A* in stomach adenocarcinoma (STAD) (Fig. 1a). The FAM120A protein levels in 6 pairs of fresh GC tissues and adjacent normal tissues were examined, and a higher FAM120A protein level was found in GC tissues (Fig. 1b). Moreover, immunohistochemical (IHC) staining of FAM120A was performed to assess its expression in 87 pairs of GC tissues and adjacent normal tissues (Fig. 1c). The results revealed a significantly higher protein level of FAM120A in GC tissues compared to adjacent normal tissues. Similarly, the FAM120A mRNA level was also found to be upregulated in GC tissues (Fig. 1d). To reveal the clinical significance of *FAM120A* in GC, the correlation between the *FAM120A* level and clinicopathological features was assessed. Upregulated *FAM120A* levels significantly correlated with tumor size (*P* = 0.01) and TNM stage (*P* = 0.001) (Supplementary Table 1). Moreover, GC patients with upregulated *FAM120A* levels had a poor prognosis (log-rank *P* = 0.02, Fig. 1e). Similarly, as verified by the KM plotter database, GC patients with increased FAM120A levels showed a shorter survival time (log-rank *P* = 4.3e-07, Fig. 1f). Based on univariate analyses, tumor size (*P* = 0.024), TNM stage (*P* = 0.041) and *FAM120A* levels (*P* = 0.006) were correlated with the overall survival (OS) of GC patients (Fig. 1g). Multivariate Cox regression analysis showed that *FAM120A* (*P* = 0.022) was an independent prognostic factor for the OS of GC patients. In short, our results demonstrated that FAM120A is increased in GC and can serve as a valuable predictive biomarker for OS.

To investigate *FAM120A* function in vitro, we examined *FAM120A* levels in GC cell lines (AGS, HGC27, MKN45, and SGC-7901) and a nonmalignant cell line (GES-1). *FAM120A* mRNA and protein levels were higher in GC cell lines than in GES-1 cells (Supplementary Fig. 1a, b). Among these four GC cell lines, both *FAM120A* mRNA and protein levels were highest in AGS cells and lowest in SGC-7901 cells. Then, we inhibited FAM120A expression by two shRNAs for FAM120A in AGS cells and overexpressed *FAM120A* in SGC-7901 cells (Supplementary Fig. 1c, d). The results of the CCK-8 assay showed that FAM120A depletion significantly reduced the viability of AGS cells and that FAM120A overexpression markedly increased the viability of SGC-7901 cells (Fig. 1h, i). The results of EdU staining indicated that FAM120A depletion restrained proliferation and that FAM120A overexpression facilitated the proliferation of GC cells (Fig. 1j, k). In addition, as indicated by clone formation assays, FAM120A-depleted GC cells showed a smaller number of clones, whereas GC cells overexpressing FAM120A showed more clones (Fig. 1l–n). Altogether, these data suggest that FAM120A promotes the survival and growth of GC cells.

### FAM120A is implicated in cisplatin resistance in GC by regulating ferroptosis

**Fig. 2:**
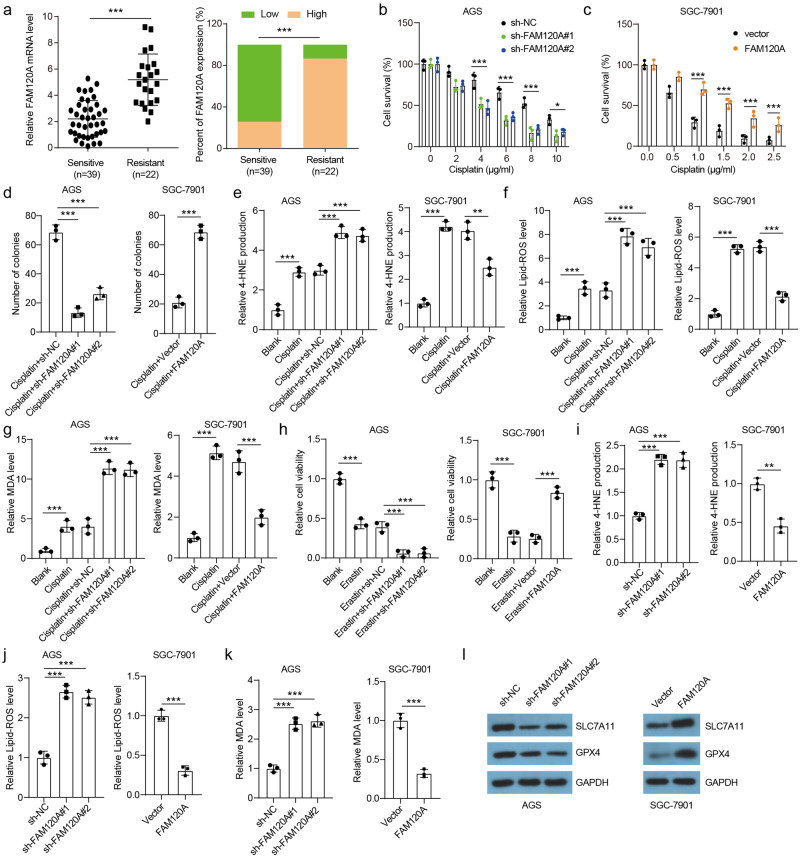
FAM120A promoted cisplatin resistance by inhibiting ferroptosis. **a** FAM120A expression in cisplatin-sensitive and cisplatin-resistant tumors in the in-house cohort. **b** FAM120A depletion reduced the resistance of AGS cells to cisplatin (*N* = 3). **c** FAM120A overexpression reduced the sensitivity of SGC-7901 cells to cisplatin (*N* = 3). **d** A colony formation assay was used to assess the sensitivity of FAM120A-depleted AGS cells and FAM120A-overexpressing SGC-7901 cells to cisplatin (*N* = 3). **e** 4-HNE production in FAM120A-depleted AGS cells and FAM120A-overexpressing SGC-7901 cells treated with cisplatin (AGS for 4 µg/ml, SGC-7901 for 0.5 µg/ml) (*N* = 3). **f** Lipid ROS were detected using C11-BODIPY in FAM120A-depleted AGS cells and FAM120A-overexpressing SGC-7901 cells treated with cisplatin (*N* = 3). **g** MDA production in FAM120A-depleted AGS cells and FAM120A-overexpressing SGC-7901 cells treated with cisplatin (*N* = 3). **h** Cell viability of GC cells with FAM120A depletion or overexpression treated with erastin (20 μM) for 48 h (*N* = 3). **i**–**k** 4-HNE, lipid ROS, and MDA levels in FAM120A-depleted AGS cells and FAM120A-overexpressing SGC-7901 cells (*N* = 3). **l** SLC7A11 and PGX4 protein levels in FAM120A-depleted AGS cells and FAM120A-overexpressing SGC-7901 cells (*N* = 3). Statistical significance was determined using Student’s *t*-test (**a**, **c**, **d**-right, **i**-right, **j**-right, **k**-right), and one-way ANOVA followed by Tukey multiple comparisons (**b**, **d**-left, **e**, **f**, **g**, **h**, **i**-left, **j**-left, **k**-left). **p < 0.01, ***p < 0.001.

Although cisplatin-based chemotherapy is recommended for patients with advanced GC, chemoresistance severely limits the prognosis of GC patients^18,19^. Here, we found that FAM120A expression was higher in cisplatin-resistant tumors than in cisplatin-sensitive tumors (Fig. 2a), indicating that FAM120A may be implicated in chemoresistance. FAM120A-depleted cells were more sensitive to cisplatin, whereas FAM120A overexpression in SGC-7901 cells decreased sensitivity to cisplatin (Fig. 2b, c). Similar results were also displayed by the clone formation assay (Fig. 2d). In addition, we found that AGS cells were more resistant than SGC-7901 cells to cisplatin. In short, our data indicate that FAM120A is increased in cisplatin-resistant GC tissues and that FAM120A depletion enhances their sensitivity to cisplatin.

Ferroptosis is a regulated mechanism of cell death induced by an iron-dependent accumulation of lipid peroxides during chemotherapy^9^. Our data suggested that cisplatin treatment significantly enhanced 4-HNE production, lipid ROS, and MDA levels in SGC-7901 and AGS cells (Fig. 2e–g), indicating ferroptosis induction by cisplatin. Moreover, FAM120A depletion in AGS cells increased 4-HNE production, lipid ROS, and MDA levels induced by cisplatin, indicating that FAM120A depletion deteriorated cisplatin-induced ferroptosis. FAM120A overexpression in SGC-7901 cells rescued the increased 4-HNE production, lipid ROS, and MDA levels induced by cisplatin, indicating that FAM120A overexpression reversed cisplatin-induced ferroptosis. In summary, ferroptosis could be triggered by cisplatin in GC cells, and FAM120A depletion sensitizes GC cells to cisplatin-induced ferroptosis in vitro.

We further asked whether FAM120A could inhibit ferroptosis universally, not only in cisplatin-induced ferroptosis. To address this question, erastin, a well‐known inducer of ferroptosis, was used to treat GC cells. The results indicated that erastin-induced ferroptosis was further enhanced by FAM120A depletion and alleviated by FAM120A overexpression (Fig. 2h). Moreover, FAM120A depletion increased 4-HNE production, lipid ROS, MDA levels, and the expression of ferroptosis-related proteins (SLC7A11 and GPX4) in AGS cells (Fig. 2i–l). FAM120A overexpression had the opposite effect. Thus, the results indicate that FAM120A acts as an inhibitor of ferroptosis and promotes cisplatin resistance.

### METTL3-induced m6A modification increases FAM120A expression

**Fig. 3:**
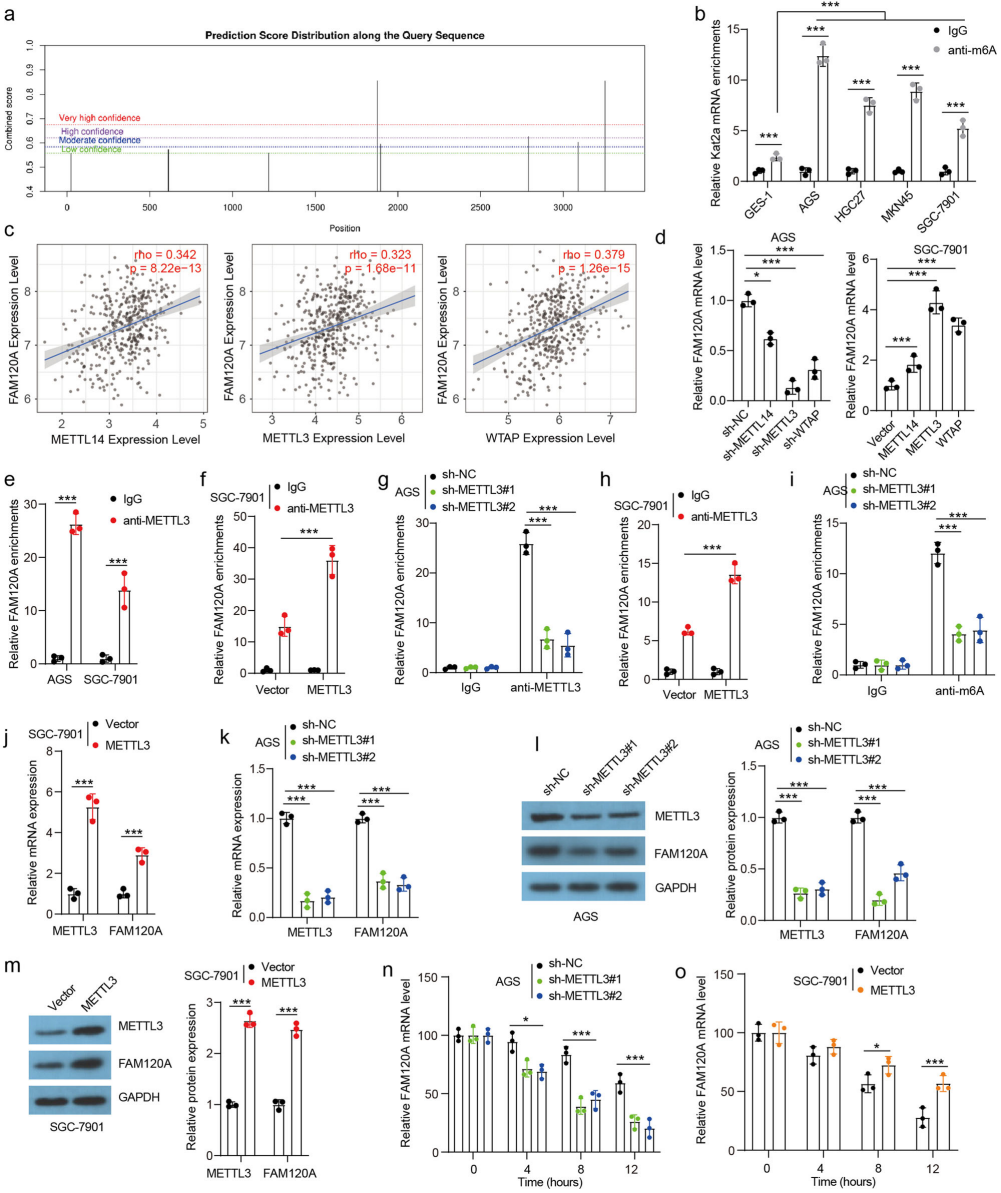
METTL3 promoted FAM120A expression by facilitating m6A modification. **a** The predicted modification sites for m6A in *FAM120A* mRNA. **b** The enrichment of m6A modifications in GES-1 cells and GC cell lines was measured by m6A-RIP assay (*N* = 3). **c** Correlation analysis of FAM120A and METTL14, METTL3, and WTAP in STAD was evaluated based on publicly available TCGA databases. **d** The *FAM120A* mRNA level in AGS and SGC-7901 cells after METTL14, METTL3 and WTAP depletion or overexpression (*N* = 3). **e** The binding between METTL3 and *FAM120A* mRNA in AGS and SGC-7901 cells was verified by RIP assay (*N* = 3). **f**, **g** The effect of METTL3 overexpression or knockdown on the binding between METTL3 and *FAM120A* mRNA (*N* = 3). **h**, **i** The effect of METTL3 overexpression or knockdown on m6A modification of *FAM120A* mRNA (*N* = 3). **j–m** The effect of METTL3 overexpression or depletion on the mRNA and protein levels of METTL3 and FAM120A in GC cells (*N* = 3). **n**, **o** *FAM120A* mRNA degrades rapidly in METTL3-depleted AGS cells and degrades slowly in METTL3-overexpressing SGC-7901 cells (*N* =  3). Transcription was inhibited by 5 μg/ml AcTD for the indicated time, and *FAM120A* mRNA levels were determined by RT–qPCR. Statistical significance was determined using one-way ANOVA followed by Tukey multiple comparisons (**b**, **d**, **g**, **i**, **k**, **n**), and Student’s *t*-test (**e**, f, **h**, **j**, **m**, **o**). *p < 0.05, ***p < 0.001.

N6-methyladenosine (m6A) was reported to be involved in a variety of disease processes, including tumors, by regulating RNA transcription splicing, transport, translation, and degradation^20^. We then wanted to determine whether the high expression of FAM120A in GC tissues was associated with its m6A modification. By analyzing the sequence of *FAM120A* mRNA, multiple sites for m6A were predicted by SRAMP (sequence-based RNA adenosine methylation site predictor) (Fig. 3a). We determined the m6A abundance in the *FAM120A* mRNA by m6A RNA immunoprecipitation in GES-1 cells and GC cell lines (Fig. 3b). The m6A-specific antibody significantly enriched *FAM120A* mRNA in GES-1 cells and GC cell lines. Moreover, the level of m6A-modified FAM120A mRNA was increased in GC cell lines compared with GES-1 cells.

We then explored the mechanism of the m6A modification of *FAM120A* mRNA. WTAP, METTL3, and METTL14 are the catalytic core of the methyltransferase complex, which is important for m6A modification^21^. *FAM120A* expression showed a positive correlation with *WTAP*, *METTL3*, and *METTL14* levels in the TCGA-STAD database (Fig. 3c). WTAP and METTL3 expression was increased in STAD (Supplementary Fig. 2a–c). GC patients with high *METTL3* expression had a poor outcome, and those with low *WTAP* expression had a poor outcome (Supplementary Fig. 2d–f). Additionally, an upregulation of *METTL3* was also observed in cisplatin-resistant tumors, while there were no significant differences detected in the expression levels of *WTAP* and *METTL14* between cisplatin-resistant and cisplatin-sensitive tumors (Supplementary Fig. 2g–i). Moreover, WTAP, METTL3, and METTL14 depletion significantly reduced FAM120A mRNA levels in AGS cells, and the inhibitory effect of METTL3 deletion on *FAM120A* mRNA expression was most obvious (Fig. 3d). WTAP, METTL3, and METTL14 overexpression increased the expression of *FAM120A* mRNA, and the facilitation was most obvious after METTL3 overexpression. Thus, we next focused on exploring the role of METTL3 in post-transcriptional modification of *FAM120A*. We immunoprecipitated METTL3 from the cytoplasmic extract of GC cells and analyzed the RNAs that bind to METTL3. We observed a significantly higher METTL3 enrichment with *FAM120A* mRNA relative to IgG control (Fig. 3e), indicating that METTL3 could interact with *FAM120A* mRNA. Overexpression of METTL3 enhanced this enrichment in SGC-7901 cells, while silencing METTL3 resulted in lower enrichment with *FAM120A* mRNA in AGS cells (Fig. 3f, g). To explore whether FAM120A is modulated in an m6A-dependent manner, the m6A-specific antibody was used for RIP assay. A decrease in m6A-modified *FAM120A* mRNA was observed upon METTL3 disruption in AGS cells and METTL3 overexpression increased the levels of m6A-modified *FAM120A* mRNA in SGC-7901 cells (Fig. 3h, i). In addition, METTL3-overexpressing SGC-7901 cells showed higher mRNA and protein expression of METTL3 and FAM120A, whereas METTL3-depleted AGS cells showed lower mRNA and protein expression of METTL3 and FAM120A (Fig. 3j–m). To check whether m6A modification regulates FAM120A mRNA stability, an RNA stability assay was carried out. *FAM120A* mRNA displayed a shorter half-life in METTL3-depleted cells (Fig. 3n) and a longer half-life in METTL3-overexpressing cells (Fig. 3o), indicating that the m6A modification of *FAM120A* mRNA increased its stability. Thus, our data suggest that the m6A modification of *FAM120A* mRNA induced by METTL3 promotes FAM120A expression by enhancing its stability.

### YTHDC1 protects m6A-modified FAM120A mRNA

**Fig. 4:**
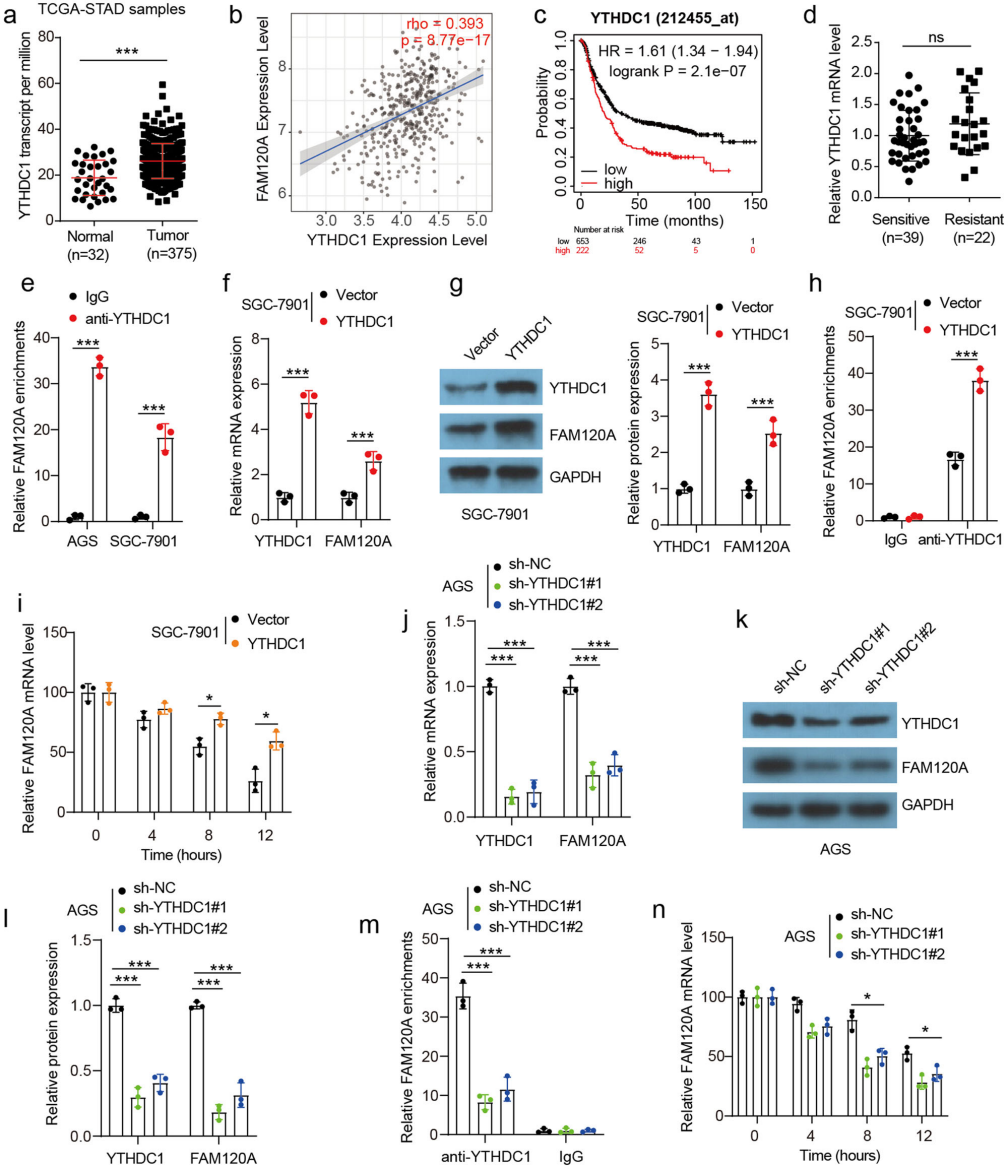
YTHDC1 enhanced the stability of m6A-modified FAM120A. **a** The YTHDC1 level in STAD was evaluated based on the publicly available TCGA database. **b** Correlation analysis of FAM120A and YTHDC1 in STAD was evaluated based on publicly available TCGA databases. **c** Kaplan‒Meier survival curve of the YTHDC1 level was obtained from the publicly available KM plotter database. **d** The expression of YTHDC1 in cisplatin-sensitive and cisplatin-resistant tumors in the in-house cohort. **e** The binding between YTHDC1 and *FAM120A* mRNA in AGS and SGC-7901 cells was verified by RIP assay. **f**, **g** The mRNA and protein levels of YTHDC1 and FAM120A in YTHDC1-overexpressing SGC-7901 cells (*N* = 3). **h** The effect of YTHDC1 overexpression on the binding between YTHDC1 and *FAM120A* mRNA in SGC-7901 cells. **i** *FAM120A* mRNA degrades slowly in YTHDC1-overexpressing SGC-7901 cells. **j**–**l** The mRNA and protein levels of YTHDC1 and FAM120A in METTL3-depleted AGS cells (*N* = 3). **m** The effect of YTHDC1 depletion on the binding between YTHDC1 and *FAM120A* mRNA in AGS cells. **n** *FAM120A* mRNA degrades rapidly in METTL3-depleted AGS cells. Statistical significance was determined using Student’s *t*-test (**a**, **d**, **e**, **f**, **g**, **h**, **i**), and one-way ANOVA followed by Tukey multiple comparisons (**j**, **l**, **m**, **n**). ns *p* > 0.05, *p < 0.05, ***p < 0.001.

We then explored the potential mechanism by which m6A modification maintains *FAM120A* mRNA stability. YTHDC1 is a classical m6A reader and is implicated in the nuclear export, mRNA translation, and RNA stabilization of target RNA^22^, which has rarely been reported in GC. By using the TCGA database, we found that *YTHDC1* was significantly upregulated in STAD and showed a positive correlation with *FAM120A* expression in STAD (Fig. 4a, b). GC patients with high *YTHDC1* expression showed a poor outcome (Fig. 4c). In addition, the expression levels of *YTHDC1* did not exhibit any significant differences between cisplatin-resistant and cisplatin-sensitive tumors (Fig. 4d). Compared to IgG control, increased YTHDC1 enrichment with *FAM120A* mRNA was observed in GC cells (Fig. 4e). Overexpression of YTHDC1 increased the mRNA and protein expression of YTHDC1 and FAM120A (Fig. 4f, g). YTHDC1 overexpression enhanced YTHDC1 enrichment with *FAM120A* mRNA and increased the stability of *FAM120A* mRNA (Fig. 4h, i). However, YTHDC1 depletion plays the opposite role (Fig. 4j–n). Altogether, our results indicate that YTHDC1 could bind to and enhance the stability of m6A-modified *FAM120A* mRNA.

### METTL3/YTHDC1 regulates ferroptosis and cisplatin resistance by promoting FAM120A expression

**Fig. 5:**
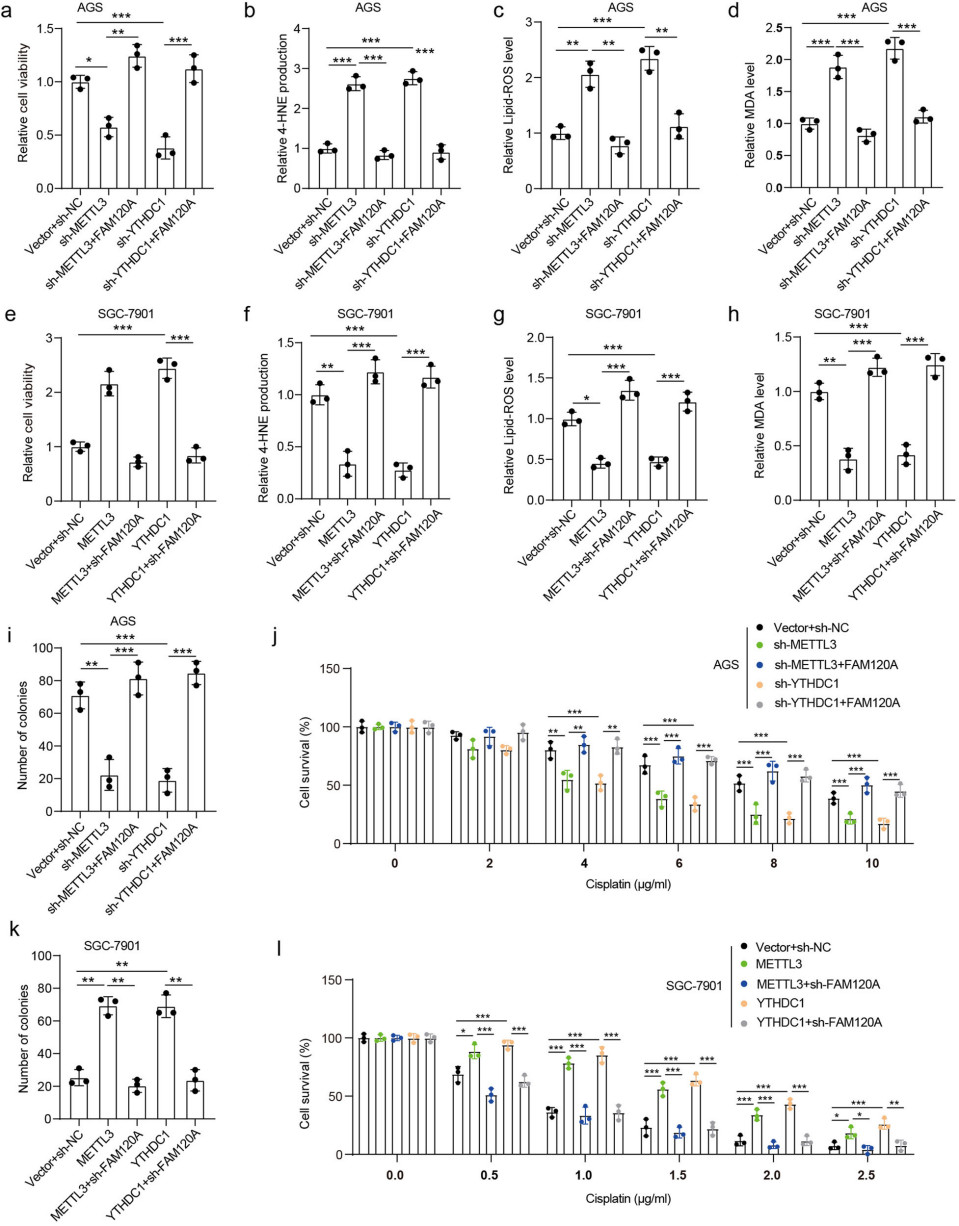
METTL3 and YTHDC1 regulated cisplatin resistance and ferroptosis in GC cells by enhancing FAM120A. Transfected GC cells were treated with cisplatin for 24 h and then cells and culture supernatants were collected. **a** Cell viability of AGS cells was measured by CCK-8. **b** 4-HNE production in AGS cells with indicated transfection. **c** Lipid peroxidation level in AGS cells with indicated transfection. **d** MDA level in AGS cells with indicated transfection. **e** Cell viability of SGC-7901 cells with indicated transfection. **f** 4-HNE production in SGC-7901 cells with indicated transfection. **g** Lipid ROS level in SGC-7901 cells with indicated transfection. **h** MDA level in SGC-7901 cells with indicated transfection. **i** Number of clones formed in AGS cells treated with cisplatin were detected by clone formation assay. **j** Cell viability of AGS cells treated with a series of concentrations of cisplatin was detected by CCK-8. **k** Number of clones formed in SGC-7901 cells treated with cisplatin was detected by clone formation assay. **l** Cell viability of SGC-7901 cells treated with a series of concentrations of cisplatin was detected by CCK-8. *N* = 3 biological replicates analyzed by one-way ANOVA followed by Tukey’s test for multiple comparisons. *p < 0.05, **p < 0.01, ***p < 0.001.

To further confirm whether METTL3 and YTHDC1 participated in the regulation of ferroptosis and cisplatin resistance by promoting FAM120A expression in GC, we re-expressed FAM120A in METTL3-depleted or YTHDC1-depleted AGS cells. The depletion of METTL3 resulted in a decrease in both mRNA and protein expression levels of METTL3 and FAM120A, while exhibiting no impact on the expression of YTHDC1 (Supplementary Fig. 3a–g). Similarly, the depletion of YTHDC1 led to a decline in the levels of both mRNA and protein expression for YTHDC1 and FAM120A, without affecting the expression of METTL3. The re-expression of FAM120A rescued the reduction in FAM120A levels caused by the depletion of METTL3 or YTHDC1. METTL3 or YTHDC1 depletion inhibited cell viability and increased 4-HNE production, lipid ROS levels, and MDA production in AGS cells treated with cisplatin (Fig. 5a–d), suggesting that METTL3 or YTHDC1 depletion promoted cisplatin-induced ferroptosis. FAM120A overexpression reversed the promotion of ferroptosis induced by METTL3 or YTHDC1 depletion. We also depleted FAM120A in METTL3-overexpressing or YTHDC1-overexpressing SGC-7901 cells. Overexpression of METTL3 or YTHDC1 resulted in upregulation of both mRNA and protein levels of FAM120A, while depletion of FAM120A rescued its expression in SGC-7901 cells overexpressing METTL3 or YTHDC1 (Supplementary Fig. 3h–m). METTL3 or YTHDC1 overexpression inhibited cisplatin-induced ferroptosis, and FAM120A depletion rescued the inhibition of ferroptosis induced by METTL3 or YTHDC1 overexpression (Fig. 5e–h), indicating that METTL3 or YTHDC1 was related to cisplatin-induced ferroptosis in GC cells by regulating FAM120A expression. Moreover, we further confirmed that depletion of METTL3 or YTHDC1 significantly attenuated cisplatin resistance in AGS cells, while overexpression of FAM120A restored the reduced cisplatin resistance caused by METTL3 or YTHDC1 depletion (Fig. 5i, j). Consistently, METTL3 or YTHDC1 overexpression reduced cisplatin sensitivity in SGC-7901 cells, and FAM120A depletion recovered cisplatin sensitivity reduced by METTL3 or YTHDC1 overexpression (Fig. 5k, l), indicating that METTL3 and YTHDC1 could regulate cisplatin sensitivity in GC cells by upregulating FAM120A. Together, these results demonstrate that METTL3 and YTHDC1 inhibit ferroptosis and cisplatin resistance by promoting FAM120A expression.

### FAM120A inhibits ferroptosis and promotes cisplatin resistance by stabilizing *SLC7A11* mRNA

**Fig. 6:**
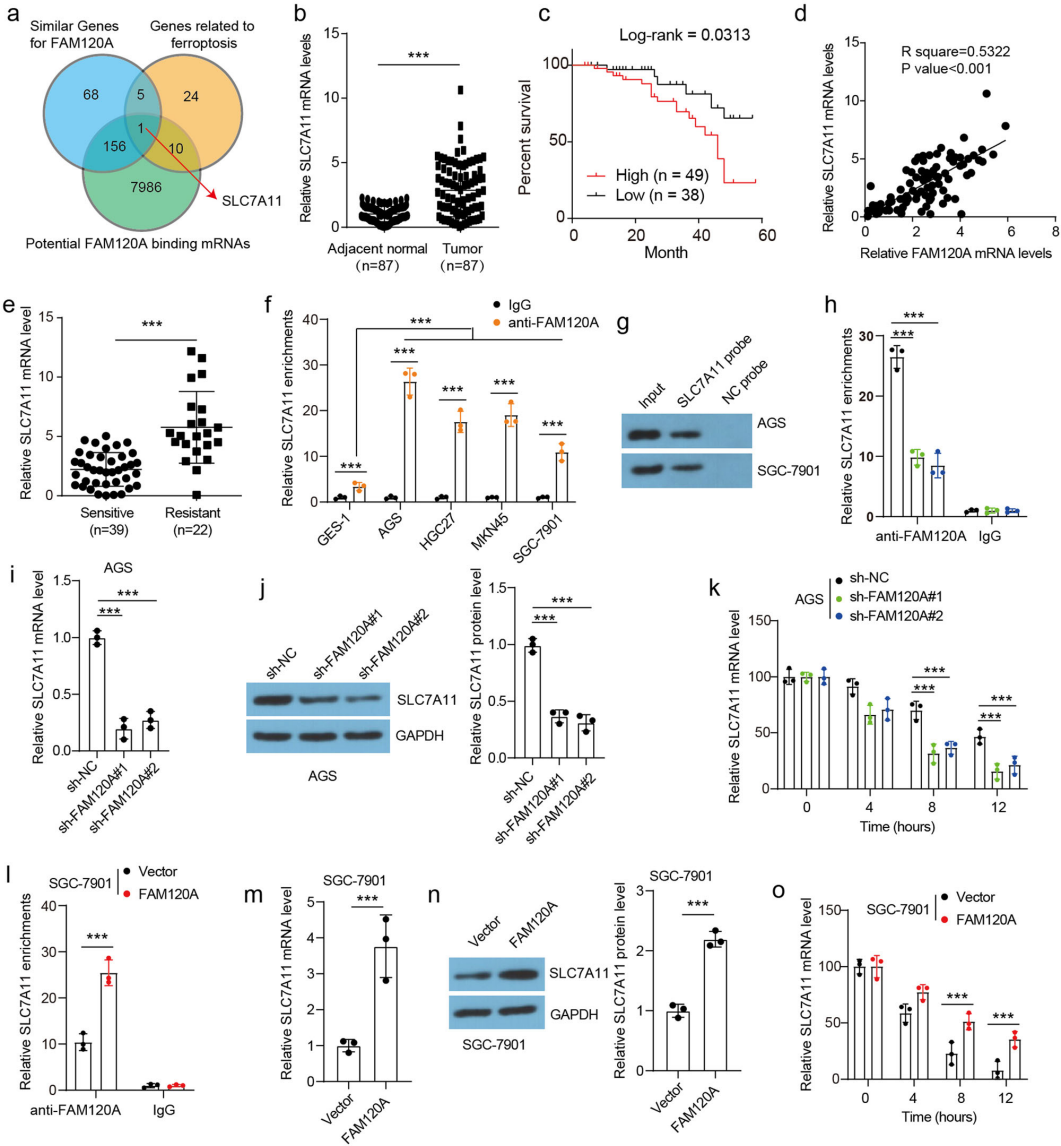
SLC7A11 was a target for FAM120A. **a** Venn diagram showing genes predicted to interact with FAM120A, similar to FAM120A, and previously reported ferroptosis-related genes. **b** SLC7A11 levels in the in-house GC and adjacent normal tissues were measured by qRT‒PCR. **c** Overall survival curve of GC patients with low and high SLC7A11 expression in the in-house cohort. **d** Correlation analysis of FAM120A and SLC7A11 in GC tissues in the in-house cohort. **e** The expression of SLC7A11 in cisplatin-sensitive and cisplatin-resistant tumors in the in-house cohort. **f** The binding between SLC7A11 mRNA and FAM120A in GES-1 and GC cell lines was verified by RIP assay (*N* = 3). **g** The binding between *SLC7A11* mRNA and FAM120A was verified by RNA pull-down assay. **h** FAM120A depletion reduced the enrichment of FAM120A with SLC7A11 mRNA in AGS cells (*N* = 3). **i**, **j** The mRNA and protein expression of SLC7A11 in FAM120A-depleted AGS cells (*N* = 3). **k** SLC7A11 mRNA degrades rapidly in FAM120A-depleted AGS cells (*N* = 3). **l** FAM120A overexpression increased the enrichment of FAM120A with *SLC7A11* mRNA in SGC-7901 cells (*N* = 3). **m** The mRNA and protein expression of SLC7A11 in FAM120A-overexpressing SGC-7901 cells (*N* = 3). **n** *SLC7A11* mRNA degrades slowly in FAM120A-overexpressing SGC-7901 cells (*N* = 3). Statistical significance was determined using Student’s *t*-test (**b**, **e**, **l**, **m**, **n**, **o**), Pearson’s chi-square test (d), and one-way ANOVA followed by Tukey multiple comparisons (**f**, **h**, **i**, **j**, **k**). ***p < 0.001.

FAM120A, a scaffold protein, has been reported to affect biological processes by interacting with related proteins or RNAs^17,23^. To identify potential downstream targets of FAM120A, the potential FAM120A binding mRNAs were predicted by ENCORI (Encyclopedia of RNA Interactomes) (Fig. 6a). Sixteen of the 40 ferroptosis-related genes overlapped and were predicted to bind FAM120A. SLC7A11 was filtered out because it is significantly correlated with FAM120A expression and is a ferroptosis-related protein predicted to interact with FAM120A. *SLC7A11* levels were found to be increased in GC tissues in our samples and TCGA-STAD databases (Fig. 6b, Supplementary Fig. 4a). GC patients with higher *SLC7A11* had a shorter overall survival time (Fig. 6c, Supplementary Fig. 4b). *SLC7A11* expression in GC tissues showed a positive correlation with *FAM120A* levels in our samples and STAD samples (Fig. 6d, Supplementary Fig. 4c). In addition, *SLC7A11* mRNA expression was higher in cisplatin-resistant tumors (Fig. 6e). To investigate the regulatory mechanisms between FAM120A and *SLC7A11*, a RIP assay was first carried out. The FAM120A-specific antibody significantly enriched *SLC7A1*1 mRNA in GES-1 cells and GC cell lines (Fig. 6f). Increased FAM120A enrichment with *SLC7A11* mRNA was observed in GC cells. The results of RNA pull-down assays further verified the binding between FAM120A and *SLC7A11* mRNA in both AGS and SGC-7901 cells (Fig. 6g). FAM120A depletion in AGS cells resulted in a reduction of FAM120A enrichment with *SLC7A11* mRNA (Fig. 6h), leading to the inhibition of both mRNA and protein expression of SLC7A11 (Fig. 6i, j), as well as a decrease in the stability of *SLC7A11* mRNA (Fig. 6k). Conversely, the overexpression of FAM120A in SGC-7901 cells resulted in an enhanced enrichment of FAM120A with *SLC7A11* mRNA (Fig. 6l), an increased expression of both mRNA and protein levels of SLC7A11 (Fig. 6m, n), and an elevated stability of *SLC7A11* mRNA (Fig. 6o). Overall, these results demonstrate that SLC7A11 is upregulated in GC and a target for FAM120A.

To further verify whether FAM120A inhibited ferroptosis and promoted cisplatin resistance by promoting SLC7A11 expression, gain- and loss-of-function assays were carried out. FAM120A depletion significantly reduced the mRNA and protein expression of FAM120A and SLC7A11 in AGS cells (Supplementary Fig. 5a, b). The re-expression of SLC7A11 in FAM120A-depleted AGS cells recovered the mRNA and protein expression of SLC7A11, without affecting the expression of FAM120A. SLC7A11 overexpression enhanced cell viability, reduced 4-HNE production, decreased lipid ROS levels, and inhibited MDA production in AGS cells treated with cisplatin (Supplementary Fig. 5c–f), indicating that SLC7A11 overexpression inhibited cisplatin-induced ferroptosis in AGS cells. SLC7A11 overexpression reversed the promotion of ferroptosis induced by FAM120A depletion. Similarly, SLC7A11 depletion reversed the inhibition of ferroptosis induced by FAM120A overexpression in SGC-7901 cells (Supplementary Fig. 5g–l), indicating that FAM120A regulated cisplatin-induced ferroptosis by enhancing SLC7A11 expression. In addition, the data from the clone formation assay and CCK-8 assay indicated that SLC7A11-overexpressing AGS cells were more resistant than control cells (Supplementary Fig. 5m, n). The sensitivity to cisplatin enhanced by FAM120A depletion was rescued by SLC7A11 overexpression. SLC7A11 depletion enhanced the sensitivity to cisplatin in SGC-7901 cells, and SLC7A11 depletion reversed cisplatin resistance induced by FAM120A overexpression (Supplementary Fig. 5o, p), suggesting that FAM120A regulated cisplatin resistance by enhancing SLC7A11 expression. Overall, FAM120A regulates ferroptosis and cisplatin resistance by increasing SLC7A11 expression.

### FAM120A deficiency promotes cisplatin sensitivity partly by enhancing ferroptosis in vivo

**Fig. 7:**
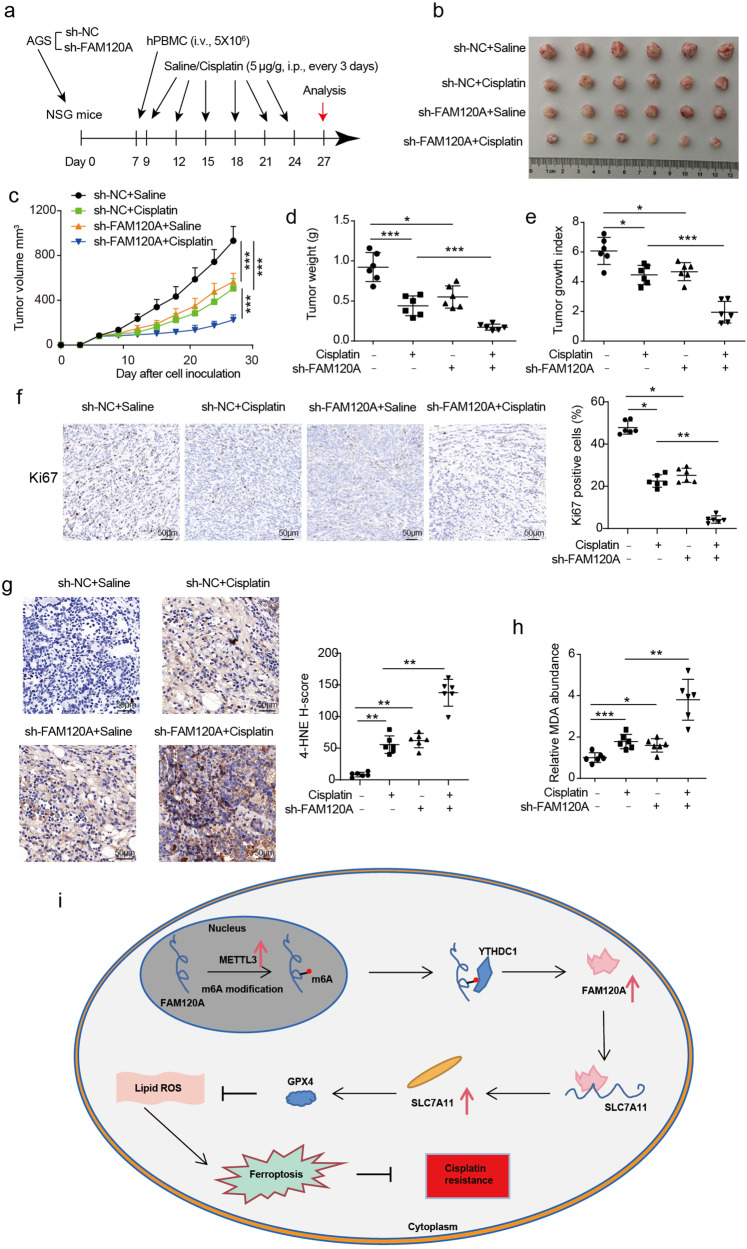
FAM120A deficiency promotes cisplatin sensitivity in vivo. **a** Schematic description of the animal experimental design. **b** Representative images of xenografts. **c** Tumor growth curves of the mouse xenograft model. **d** The tumor weight of xenografts. **e** The tumor growth index was calculated. **f** IHC staining and KI67-positive cells in xenograft sections. Scale bars = 50 μm. **g** IHC staining and H-score for 4-HNE in xenografts. Scale bars = 50 μm. **h** MDA production in tumor tissues. **i** Schematic diagram of the model proposed in this study. *N* = 6 per group analyzed by one-way ANOVA followed by Tukey’s test for multiple comparisons. *p < 0.05, **p < 0.01, ***p < 0.001.

To further explore the function of FAM120A in vivo, a xenograft GC mouse model was established by injecting AGS-sh-NC cells or AGS-sh-FAM120A cells into the axilla of NSG mice. When the average tumor size reached 100 mm^3^ (approximately Day 7 post tumor inoculation), the mice were reconstituted with human peripheral blood mononuclear cells (hPBMCs). Two days later, mice were treated with saline or cisplatin (5 μg/g, i.p., every 3 days) (Fig. 7a). Tumors derived from FAM120A-depleted cells were consistently smaller and lighter than those formed by control GC cells, indicating that FAM120A depletion inhibited tumor growth in vivo (Fig. 7b–d). In addition, cisplatin treatment significantly reduced the volumes and weights of subcutaneous tumors. To assess the combined effect of FAM120A depletion and cisplatin, we introduced the tumor growth index to normalize the variations in tumor volume between the sh-FAM120A and sh-NC groups before initial cisplatin treatment. When compared to cisplatin therapy alone, the combination of FAM120A depletion with cisplatin significantly diminished the tumor growth index in GC (Fig. 7e), indicating that FAM120A depletion enhanced sensitivity to cisplatin in vivo. As Ki67 is the most widely used marker of cell proliferation in cancer, we also detected Ki67 expressions in xenografts by IHC. Ki67-positive cells were decreased in FAM120A-depleted xenografts and cisplatin-treated xenografts, indicating that FAM120A depletion or cisplatin inhibited the proliferation of AGS cells in vivo (Fig. 7f). FAM120A depletion in combination with cisplatin therapy showed the strongest inhibition of tumor growth. To better investigate the role of ferroptosis in vivo, the slices of subcutaneous tumors were stained by 4-HNE, and the production of MDA in subcutaneous tumors was measured by ELISA (Fig. 7g, h). The induced production of 4-HNE and MDA by cisplatin was further enhanced by FAM120A depletion. Therefore, these data indicate that FAM120A depletion enhances cisplatin sensitivity in GC by enhancing ferroptosis.

## Discussion

Although the mortality of GC has decreased over the past few years, the current treatment effect is still unsatisfactory^24^. As cisplatin-based chemotherapy remains the mainstay of treatment for advanced GC, it is urgent to understand the mechanisms of cisplatin resistance and to seek effective therapeutic targets. Here, we authenticate the crucial function of METTL3-m6A-YTHDC1-FAM120A-SLC7A11-ferroptosis pathway in cisplatin resistance of GC (Fig. 7i). Our study provides a potential target for the prevention and treatment of GC patients, and targeting the METTL3-m6A-YTHDC1-FAM120A-SLC7A11 pathway is expected to improve cisplatin resistance of GC patients.

N6-methyladenosine (m6A) is an important RNA modification that is regulated by methyltransferases, demethylases, and effector proteins^25^. m6A is implicated in tumor formation and development, as well as radiotherapy, chemotherapy, and immunotherapy^26–28^. A mounting body of research indicates a close correlation between m6A and the development of chemoresistance in GC. The m6A methyltransferase KIAA1429 enhanced the cisplatin resistance of GC cells by enhancing FOXM1 expression in a m6A-YTHDF1-dependent manner^29^. The m6A demethylase FTO regulates the cisplatin resistance of GC by activating autophagy through *ULK1*^30^. Li et al. found that METTL3 promotes oxaliplatin resistance of GC CD133+ stem cells by enhancing *PARP1* mRNA stability^31^. METTL3 was also reported to regulate chemoresistance in GC by stimulating m^6^A modification of *ARHGAP5* mRNA^32^. METTL3-mediated m6A modification on ABL enhances the resistance of GC cells to chemotherapy^33^. In this study, we found METTL3 was increased in GC tissues and GC patients with high METTL3 expression had a poor prognosis. Inhibition of METTL3 in GC cells enhanced the sensitivity of GC cells to cisplatin. Mechanically, we indicated that METTL3-mediated m6A modification on *FAM120A* mRNA enhanced FAM120A expression in an m6A-YTHDC1-dependent manner, which further promoted cisplatin resistance of GC cells. Our results further affirmed the role of METTL3 in chemoresistance of GC, demonstrated its regulatory network, and laid a solid foundation for METTL13 targeted therapy in GC. Our study further strengthens the link between m6A and chemoresistance in GC.

YTHDC1 serves as the primary nuclear reader protein, which interacts with m6A to control mRNA splicing, exportation, and stability^34^. However, the specific role and corresponding mechanism of YTHDC1 in GC are still unclear. In this study, we first revealed the role of YTHDC1 in GC. YTHDC1 was found increased in GC tissues and associated with the prognosis of GC. YTHDC1 could bind to m6A-modified *FAM120A* mRNA and enhance its stability. YTHDC1-depleted GC cells were more sensitive to cisplatin. However, the role of YTHDC1 in different cancer types remains a subject of debate among researchers studying m6A readers. YTHDC1 is reported to promote leukemogenesis^35^, lung metastasis of triple-negative breast cancer^36^, and enhance the malignant progression in head and neck squamous cell carcinoma^37^. YTHDC1 inhibits the progression of renal cancer^34^, glioma^38^, lung cancer^39^ and pancreatic cancer^40^. The potential variation in the functionality of YTHDC1 across tumors could potentially be attributed to its varying expression patterns within specific tumor types and its influence on unique sets of downstream target genes.

FAM120A, serving as a scaffold protein, is reported to promote colon cancer metastasis^23^ and regulate the growth and invasion of GC cells^17^. Similar to the results of Tanaka et al.^17^, we also confirm that FAM120A functions as a proto-oncogene in GC. Increased FAM120A expression also is found in the in-house GC tissues and the depletion of FAM120A inhibited the growth of GC in vitro and in vivo. More importantly, we found that FAM120A is closely related to drug resistance in GC. Upregulated FAM120A level is observed in cisplatin resistant GC tissues and FAM120A overexpression increases the cisplatin resistance of GC cells. As the increased METTL3 expression and unchanged YTHDC1 expression in cisplatin-resistant tumors, we speculate that the increased FAM120A in resistant GC may be primarily caused by the increased METTL3. The increased METTL3 expression in resistant tumor cells enhances the m6A modification of *FAM120A* mRNA. Then, YTHDC1 enhanced the stability of *FAM120A* mRNA in an m6A-dependent manner, leading to the upregulation of FAM120A in resistant tumor cells. Our study revealed the function of FAM120A in chemotherapy tolerance. Targeting FAM120A is an effective strategy to alleviate cisplatin resistance in GC.

FAM120A, acting as an RNA binding protein, can directly bind to *IGF-II* mRNA and promotes the extracellular secretion of IGF-II protein in GC cells^17^. In this study, we identify another target for FAM120A in GC. We found that FAM120A can bind to S*LC7A11* mRNA and enhance its stability to inhibit ferroptosis in GC cells. FAM120A also is involved in the regulation of adipogenesis by regulating lipogenic gene expression^41^. Van Acker et al. indicated that the binding ratios of neuroglobin and FAM120A are increased under ferroptosis stress^42^, indicating that FAM120A may be involved in ferroptosis in neuroblastoma. In our study, we show that FAM120A regulates ferroptosis. FAM120A knockdown accelerated cisplatin-induced ferroptosis by enhancing lipid peroxidation. The present study would significantly expand our understanding of the functional and regulatory network associated with FAM120A in GC.

Ferroptosis can be triggered by several cancer treatments, such as chemotherapy, radiotherapy, immunotherapy, and targeted therapies^43–45^. Studies have shown that ferroptosis is implicated in cisplatin resistance in GC. Fu et al. suggested that cisplatin-resistant GC cells exhibited lower ferroptosis and ATF3-induced ferroptosis alleviated cisplatin resistance in GC by inhibiting Nrf2/Keap1/xCT pathway^15^. Similarly, Wang et al. indicated that TCF4 deficiency promoted cisplatin-induced ferroptosis in vitro and in vivo in GC^13^. Besides, cisplatin promoted miR-522 secretion from CAFs, leading to ALOX15 suppression and inhibited ferroptosis, and ultimately resulted in promoted acquired chemo-resistance^14^. CircHIPK3 was reported to promote cisplatin resistance in GC by blocking autophagy-dependent ferroptosis^46^. In addition to cisplatin resistance in GC, ferroptosis is also involved in cisplatin resistance in ovarian cancer^47^, head and neck cancer^48^, and non-small-cell lung cancer^49^. In this study, we elucidated METTL3-m6A-YTHDC1-FAM120A-SLC7A11-ferroptosis signaling pathway involved in the regulation of cisplatin resistance in GC. Our study further demonstrated the important role of ferroptosis in cisplatin resistance in GC, suggesting that inhibition of ferroptosis may be an effective strategy to alleviate cisplatin resistance in GC.

In summary, our study identified FAM120A as a tumor-promoting factor that plays an important role in promoting cisplatin resistance in GC by inhibiting ferroptosis. We noted that METTL3-m6A-YTHDC1 induced an increase in FAM120A-inhibited cisplatin resistance by activating SLC7A11-mediated ferroptosis. These results illustrate a promising strategy for alleviating cisplatin resistance in GC.

## Methods

### Patient samples

A total of 87 GC tissues and their matched normal tissues were collected from patients who did not receive radiotherapy or chemotherapy enrolled in the Sichuan Provincial People’s Hospital between 2009 and 2017. Clinicopathologic features are summarized in Supplementary Table 1. In addition, 6 fresh GC tissues and paired adjacent normal tissues were also collected at the Sichuan Provincial People’s Hospital between 2021 and 2022 for Western blotting analysis. Written informed consent was acquired from all patients who participated in the study. The Ethics Committee of Sichuan Provincial People’s Hospital approved the study. All ethical regulations relevant to human research participants were followed.

### Cell culture and transfection

The normal gastric epithelial cell line GES-1 and GC cell lines (AGS, HGC27, MKN45, and SGC-7901) were purchased from the Cell Bank of the Chinese Academy of Science (Shanghai, China). All cell lines were incubated with RPMI 1640 medium (Solarbio, Beijing, China) supplemented with 10% FBS (Solarbio) in a humidified incubator at 37 °C containing 5% CO2.

FAM120A, WTAP, METTL3, METTL14, YTHDC1, SLCTA11, and PD-L1 knockdown and overexpression lentiviruses and their respective control vectors were generated by GenePharma (Shanghai, China). GC cells were inoculated into 6-well dishes and then infected with lentivirus at 60% confluence. Stable transfected cells were produced by treating cells with puromycin (4 μg/ml) for 2 weeks. The shRNA sequences are listed in Supplementary Table 2.

### Western blotting

RIPA lysis buffer (Beyotime) supplemented protease inhibitors were used to extract total protein from cells or tissues. After the determination of protein concentration using the BCA Protein Assay kit (Beyotime), proteins were further separated by SDS-PAGE and transferred to PVDF membranes (Millipore, Bedford, MA, USA). After blocking with 5% nonfat milk for 2 h, the membranes were then incubated with primary antibodies and secondary antibodies. Next, an ECL detection reagent (Beyotime) was used to detect the signals. Antibodies used in this study were listed in Supplementary Table 3.

### Real-time quantitative polymerase chain reaction (RT-QPCR)

Total RNA was extracted by Trizol reagent (Beyotime) and then cDNA was generated by BeyoRT™II First Strand cDNA Synthesis Kit (Beyotime). QPCR was performed using BeyoFast™ SYBR Green One-Step RT-QPCR Kit (Beyotime). GAPDH served as an internal control and relative gene expression was calculated using the 2^−ΔΔCt^ method. The used primers for RT-QPCR assays are listed in Supplementary Table 4.

### Cell viability

The transfected cells (2 × 10^3^) were plated on a 96-well plate. Cell viability was measured with Cell Counting Kit-8 (CCK-8; Beyotime) at the indicated times following the protocol. Briefly, 10 μL of CCK-8 solution was added to each well and incubated at 37 °C for 1 h. The optical density (OD) was measured at 450 nm using a microplate reader.

### 5-Ethynyl-2’-deoxyuridine (EDU)

After transfection for 24 h, the cells were incubated with 50 μM EDU (RIBOBIO, Guangzhou, China) for 2 h and stained with DAPI. The number of EDU-positive cells was counted.

### Colony formation assay

The transfected cells were seeded into 6-well plates at a density of 1000 cells per well and maintained for 2 weeks. After being fixed with 4% paraformaldehyde, the cells were stained with Giemsa (Beyotime). Next, the colonies were imaged and counted.

### Lipid ROS assay

After indicated treatment, 10 μM BODIPY-581/591 C11 (D3861, Thermo Fisher Scientific) was added to cells and incubated for 30 min. Next, lipid ROS were measured in a Bio-Tek fluorescence microplate reader (Winooski, VA).

### MDA assay

The relative MDA concentration in cell or tumor lysates was assessed using a Lipid Peroxidation (MDA) Assay Kit (ab118970, Abcam), according to the manufacturer’s instructions.

### Enzyme-linked immunosorbent assay (ELISA)

Levels of 4-HNE (4-Hydroxynonenal) in the supernatants were measured by corresponding ELISA assay kit according to the manufacturer’s protocol. A human 4-HNE (4-Hydroxynonenal) ELISA Kit (EH2080, FineTest, Wuhan, China) was used for 4-HNE detection.

### RIP-qPCR assay

RIP assay was carried out using Magna RIP RNA-Binding Protein Immunoprecipitation Kit (Millipore) according to the manufacturer’s protocol. Briefly, after centrifugation, the supernatant of cell lysate was inoculated with primary antibodies-conjugated beads overnight at 4 °C. Next, the binding RNAs were purified and analyzed by RT-QPCR. The primary antibodies including anti-N6-methyladenosine (m6A) (ab286164, Abcam), anti-METTL3 (ab195352, Abcam), Anti-YTHDC1 (ab220159, Abcam), Anti-FAM120A (ab156695, Abcam) and IgG (Abcam) were used.

### RNA stability assays

GC cells after indicated transfection for 24 h were treated with 5 μg/ml actinomycin D (AcTD; A1410, Sigma-Aldrich) for 0, 4, 8, or 12 h. Total RNAs were extracted and analyzed by RT-QPCR.

### RNA-pulldown assay

Biotin-labeled SLC7A11 and its antisense RNA were transcribed in vitro with the Biotin RNA Labeling Mix and T7 RNA polymerase (Roche Diagnostics, USA). After purification, biotinylated RNAs were mixed with streptavidin agarose beads (Invitrogen, USA) at 4 °C overnight. The total cell lysates from AGS or MGC-7901 cells were added to each tube for 2 h at 4 °C. After washing, the enriched proteins were detected by Western blotting.

### Humanized mouse tumor models

M-NSG (NOD-*Prkdc*^scid^*Il2rg*^em1/*Smoc*^) mice (5–6 weeks old) were obtained from the Shanghai Model Organisms Center Inc. (NM-NSG-001) and fed under special pathogen-free (SPF) conditions. Experiments in mice were approved by the Laboratory Animal Ethics Committee of the University of Electronic Science and Technology of China. We have complied with all relevant ethical regulations for animal use. The mice were randomly divided into four groups as follows: sh-NC+Saline, sh-NC+Cisplatin, sh-FAM120A+Saline, and sh-FAM120A+Cisplatin. A total of 5 × 10^6^ FAM120A-depleted or control AGS cells were subcutaneously injected into the axilla of mice. hPBMCs (5 × 10^6^ per mouse) were intravenously (I.V.) injected into NSG mice when the average tumor size reached 90 mm^3^. The width and length of the tumor were measured by Vernier calipers every 3 days, and tumor volume was calculated as follows: length × width^2^/2. All mice were sacrificed on Day 27, and the tumor weights were measured. Tumor growth index was calculated as follows: Tumor growth = (End tumor volume – Initial tumor volume)/Initial tumor volume. Initial tumor volume is defined as the time point at which treatment was started. Tumors were prepared as formalin-fixed, paraffin-embedded samples for IHC analysis.

### Histological analysis

Tissue specimens obtained from patients or mice were formalin-fixed, paraffin-embedded, and cut into 5-μm sections. Next, the sections were deparaffinized and rehydrated, followed by IHC staining as Yang et al. reported^13^. The following primary antibodies were used: anti-FAM120A (1:200; Abcam, ab229254), anti-4-hydroxynonenal (4-HNE) (1:100; Abcam, ab48506), and anti-Ki67 (1:200; Abcam, ab16667). The intensity of positive staining was scored as follows: 0 (no staining); 1 (slightly brown); 2 (moderately brown), and 3 (dark brown). The H-score was calculated by multiplying the staining intensity and the percentage of positive cells (0 to 100%).

### Statistics and Reproducibility

All data were analyzed by GraphPad Prism software (GraphPad, USA) and are presented as the mean ± SD. Sample size and replicates are stated in corresponding figure legends. Differences among two or more groups were assessed by Student’s t-test, one-way ANOVA, or two-way ANOVA. Pearson’s chi-square test was used to analyze the correlation of FAM120A expression with clinicopathological characteristics. The survival curves were analyzed by the Kaplan‒Meier method and the log-rank test. A *P* value < 0.05 was considered to indicate a statistically significant difference.

### Reporting summary

Further information on research design is available in the Nature Portfolio Reporting Summary linked to this article.

### Supplementary information

Supplementary Information

Description of Additional Supplementary Files

Supplementary Data 1

Reporting summary

## Data Availability

The authors declare that the data supporting the findings of this study are available within the paper and its supplementary information files. The source data underlying the graphs in the paper can be found in Supplementary Data 1. The Uncropped blots were shown in Supplementary Fig. 6.
