# Supplementary material for: FAM120A deficiency improves resistance to cisplatin in gastric cancer by promoting ferroptosis
Source: Commun Biol. 2024 Apr 2;7:399. doi: 10.1038/s42003-024-06097-6 (PMC10987584; doi:10.1038/s42003-024-06097-6)

Supplementary Table 1

Table S1 Correlation of FAM120A level with clinicopathologic parameters in GC patients.

**Parameters**	**Cases**	**FAM120A level**		***P*-value**
		**High**	**Low**	
Gender				
Male	57	34	23	0.36
Female	30	21	9	
Age (years)				
<61	28	17	11	0.81
≥61	59	38	21	
Tumor location				
Upper	33	20	13	0.82
Middle + lower	54	35	19	
Tumor size (cm)				
<6	31	14	17	0.01*
≥6	56	41	15	
Depth of invasion				
T1 + T2	35	18	17	0.07
T3 + T4	52	37	15	
Lymph node metastasis				
Absent	32	16	16	0.07
Present	55	39	16	
Differentiation				
Well + moderate	40	22	18	0.18
Poor + undifferentiated	47	33	14	
TNM stage				
I + Ⅱ	31	12	19	0.001***
Ⅲ + IV	56	43	13	

Supplementary Table 2

Table S2 Data of sequences for shRNA in this study.

**Gene**	**Sequences (5’-3’)**
sh-NC	GCTCTACTTCGACGACAAGAT
sh-FAM120A#1	GCATGAAGTTGCCAAGCAACT
sh-FAM120A#2	GCCAGTTCGTCAGTATGTTTA
sh-METTL3#1	GCTACCTGGACGTCAGTAT
sh-METTL3#2	GCTGCACTTCAGACGAATT
sh-WTAP	GGCAAGTACACAGATCTTAAC
sh-METTL14	GGACTTGGGATGATATTAT
sh-PD-L1	GCACATCCTCCAAATGAAAGG
sh-YTHDC1#1	TGGATTTGCAGGCGTGAAT
sh-YTHDC1#2	GCGAGATAGAGGACGTGAT
sh-SLC7A11	GCAGCTAATTAAAGGTCAA

Supplementary Table 3

Table S3 Antibodies used for Western blotting assay

Antibodies	Molecular weight	Dilution ratio	Purchase source and product number
FAM120A	121 kDa	1: 1000	Abcam, ab156695
GAPDH	37 kDa	1: 1000	CST, #2118
SLC7A11	37 kDa	1: 1000	Abcam, ab307601
GPX4	20 kDa	1:1000	Abcam, ab41787
METTL3	64 kDa	1: 1000	Abcam, ab195352
YTHDC1	100 kDa	1: 1000	Abcam, ab259990
Anti-rabbit IgG (secondary)		1: 1000	CST, #7074
Anti-mouse IgG (secondary)		1: 1000	CST, #7076

Supplementary Table 4

Table S4 Data of sequences for RT-QPCR in this study.

**Gene**	**Sequences (5’-3’)**
GAPDH	Forward: GGTGGTCTCCTCTGACTTCAA<br>Reverse: GTTGCTGTAGCCAAATTCGTTGT
FAM120A	Forward: GGCGGAGTCCAACCTATACC<br>Reverse: GAGGTCTGCCCTGAATTGCT
SLC7A11	Forward: TCTCCAAAGGAGGTTACCTGC<br>Reverse: AGACTCCCCTCAGTAAAGTGAC
METTL3	Forward: TTGTCTCCAACCTTCCGTAGT<br>Reverse: CCAGATCAGAGAGGTGGTGTAG
YTHDC1	Forward: AACTGGTTTCTAAGCCACTGAGC<br>Reverse: GGAGGCACTACTTGATAGACGA

Supplementary Figure 1

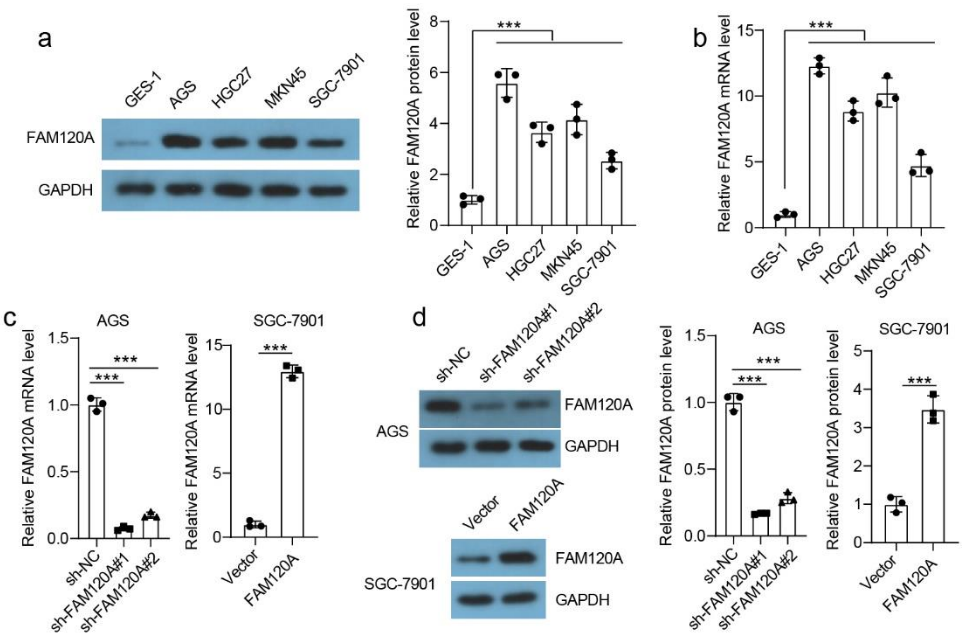

Fig. S1 FAM120A is upregulated in GC cells.

a FAM120A level in GC cell lines and GES-1 cells was detected by Western blotting. **b** FAM120A level in GC cell lines and GES-1 cells was detected RT-QPCR. **c** FAM120A level in AGS with FAM120A depletion and in MGC-7901 cells with FAM120A overexpression was detected RT-QPCR. **d** FAM120A protein level in AGS with FAM120A depletion and in MGC-7901 cells with FAM120A overexpression was detected Western blotting. $N=3$ biological replicates. Statistical significance was determined using Student's t-test (c-left, d-left), and one-way ANOVA followed by Tukey multiple comparisons (a, b, c-right, d-right). ***p<0.001.

Supplementary Figure 2

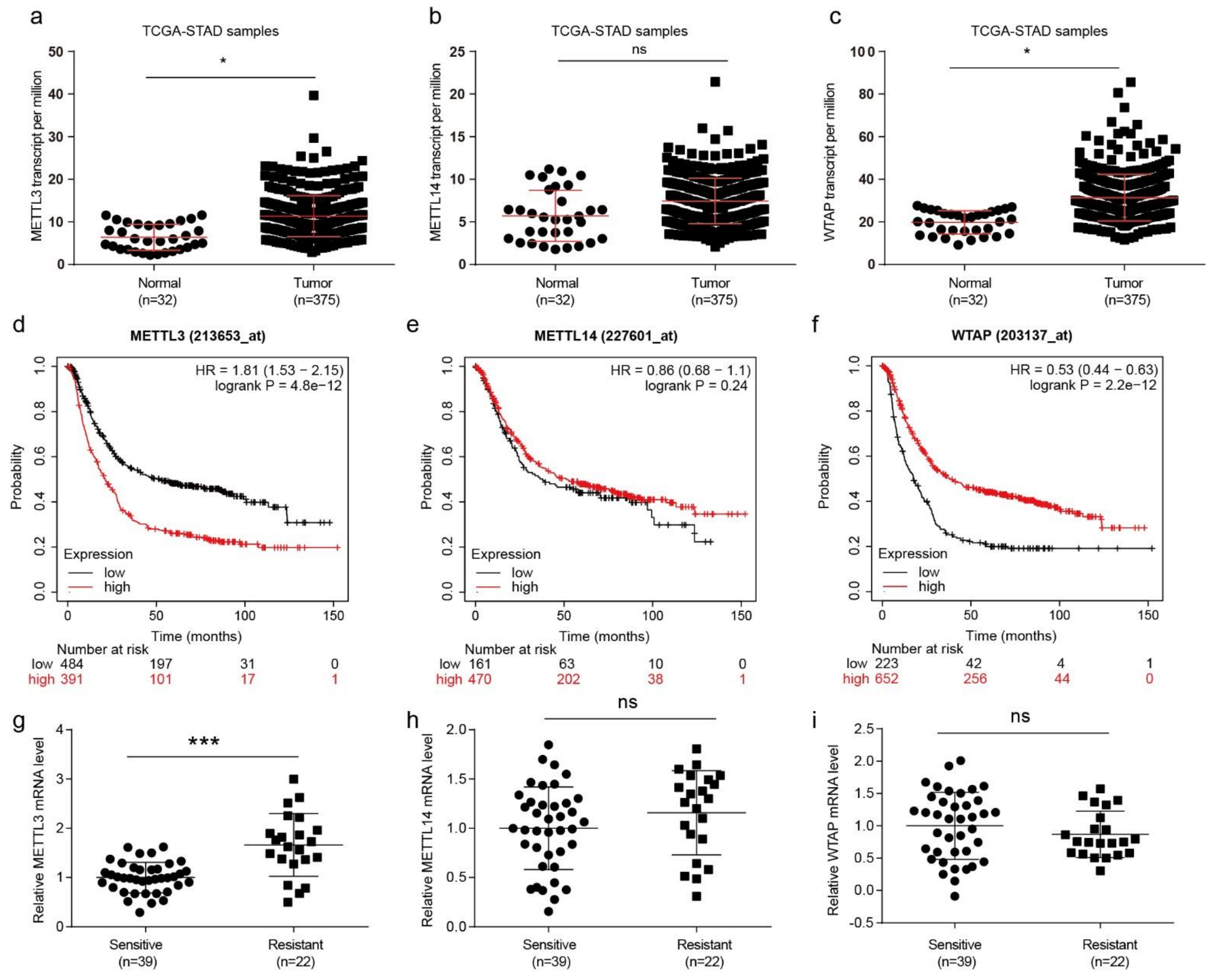

Fig. S2 METTL3 was upregulated and predicted poor prognosis in GC. a-c The expression of METTL3, METTL14 and WTAP in STAD was evaluate based on the publicly available TCGA database. **d-f** Kaplan-Meier survival curve of METTL3, METTL14 and WTAP expression was obtained from the publicly available KM plotter database. **g-i** The expression of METTL3, METTL14 and WTAP in cisplatin-sensitive and cisplatin-resistant tumors in the in-house cohort. Statistical significance was determined using Student's t-test (a, b, c, g, h, i), and the log-rank test (d, e, f). ns $p>0.05$, ***$p<0.001$.

Supplementary Figure 3

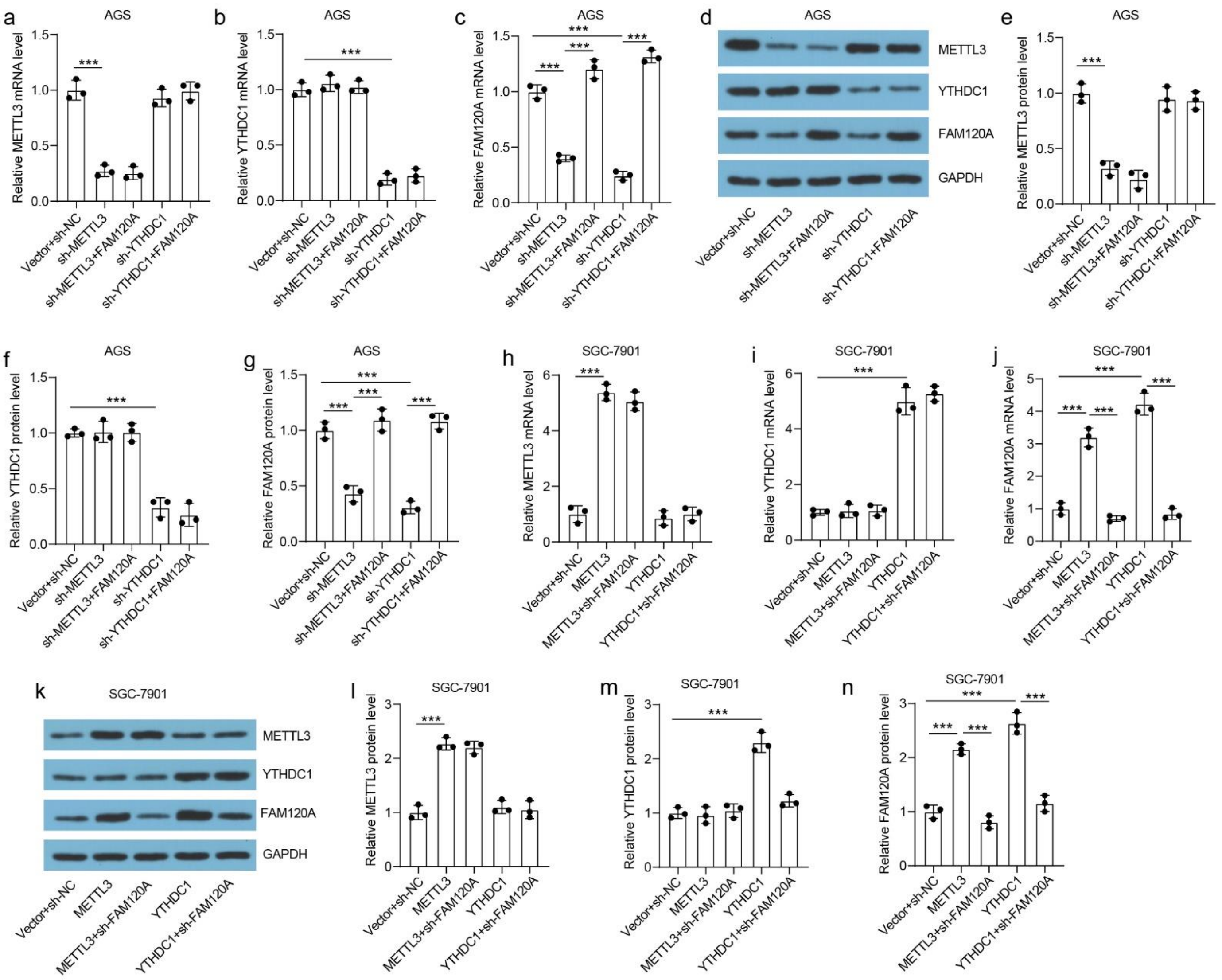

Fig. S3 METTL3 and YTHDC1 affected the expression of FAM120A in GC cells.

a-c The mRNA levels of METTL3, YTHDC1 and FAM120A in AGS cells with indicated transfection were detected by RT–QPCR. **d-g** The protein levels of METTL3, YTHDC1 and FAM120A in AGS cells with indicated transfection were detected by Western blotting. **h-j** The mRNA levels of METTL3, YTHDC1 and FAM120A in SGC-7901 cells with indicated transfection were detected by RT–QPCR. **k-n** The protein levels of METTL3, YTHDC1 and FAM120A in SGC-7901 cells with indicated transfection were detected by Western blotting. $N = 3$ biological replicates analyzed by one-way ANOVA followed by Tukey's test for multiple comparisons. *p<0.05, **p<0.01, ***p<0.001.

Supplementary Figure 4

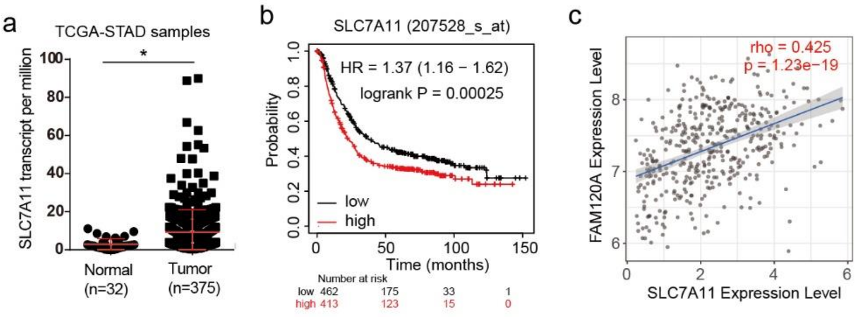

Fig. S4 SLC7A11 was upregulated in STAD.

a The expression of SLC7A11 was evaluate based on the publicly available TCGA databases. **b** Kaplan-Meier survival curve of SLC7A11 expression was obtained from the publicly available KM plotter database. **c** Correlation analysis of FAM120A and SLC7A11 in STAD was evaluate based on the publicly available TCGA databases. ***p<0.001.

Supplementary Figure 5

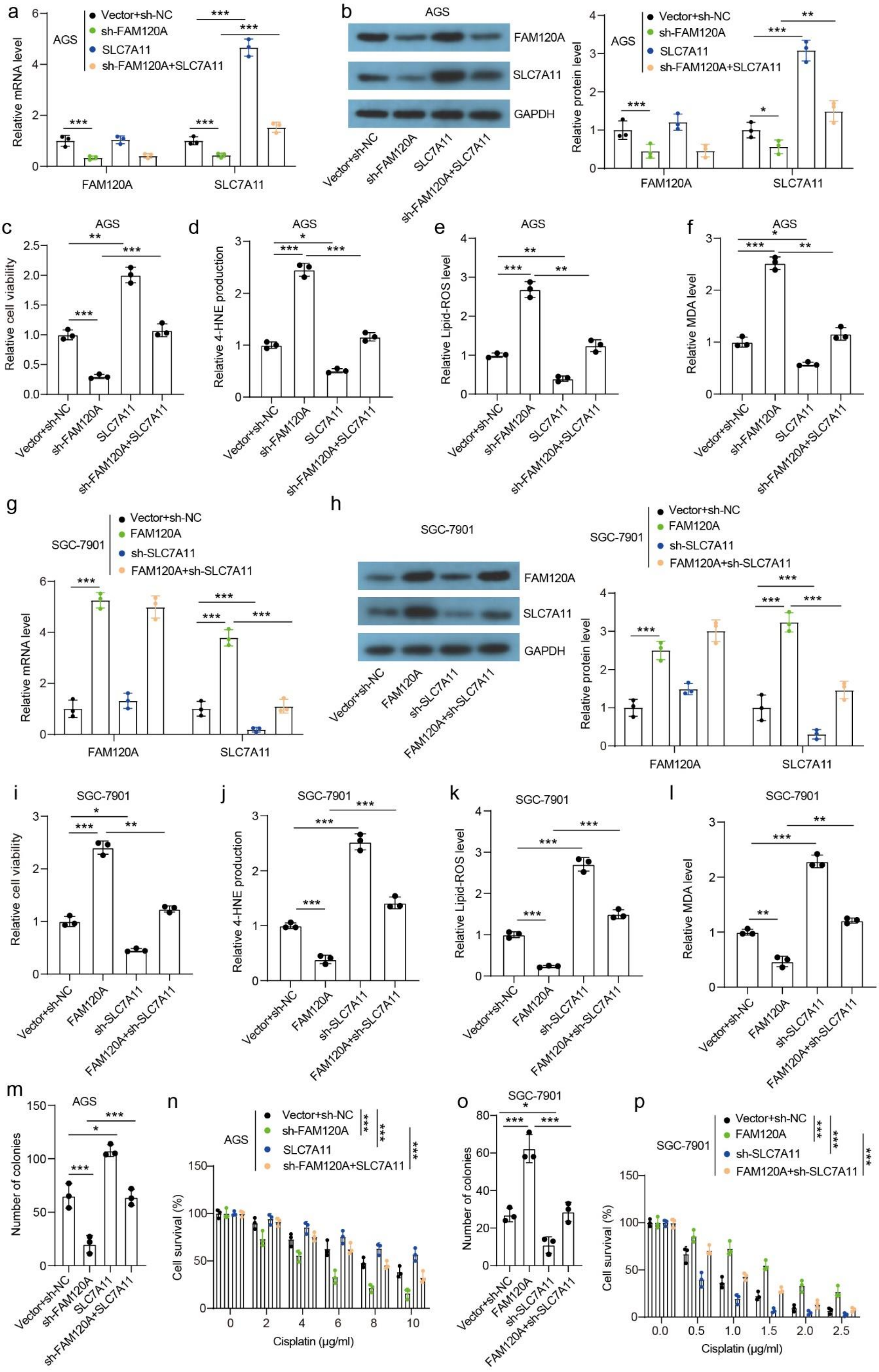

Fig. S5 FAM120A regulated cisplatin-resistance in GC cells by inhibiting SLC7A11 mediated ferroptosis. Transfected GC cells were treated cisplatin for 24 h and then cells and culture supernatants were collected.

a-b The mRNA and protein levels of FAM120A and SLC7A11 were detected in AGS cells. **c** Cell viability of AGS cells were detected. **d** 4-HNE production in AGS cells were detected. **e** Lipid ROS level in AGS cells were detected. **f** MDA level in AGS cells were detected. **g, h** The mRNA and protein levels of FAM120A and SLC7A11 were detected in SGC-7901 cells. **i** Cell viability of AGS cells were detected. **j** 4-HNE production in AGS cells were detected. **k** Lipid ROS level in AGS cells were detected. **l** MDA level in AGS cells were detected. **m** Number of clones formed in AGS cells with FAM120A depletion or SLC7A11 overexpression treated cisplatin. **n** Cell viability of AGS cells with FAM120A depletion or SLC7A11 overexpression treated with a series of concentrations of cisplatin. **o** Number of clones formed in SGC-7901 cells with FAM120A overexpression or SLC7A11 depletion treated cisplatin. **p** Cell viability of SGC-7901 cells with FAM120A overexpression or SLC7A11 depletion treated with a series of concentrations of cisplatin. $N = 3$ biological replicates analyzed by one-way ANOVA followed by Tukey's test for multiple comparisons. *p<0.05, **p<0.01, ***p<0.001.

Supplementary Figure 6: unprocessed blots

related to Figure 1b

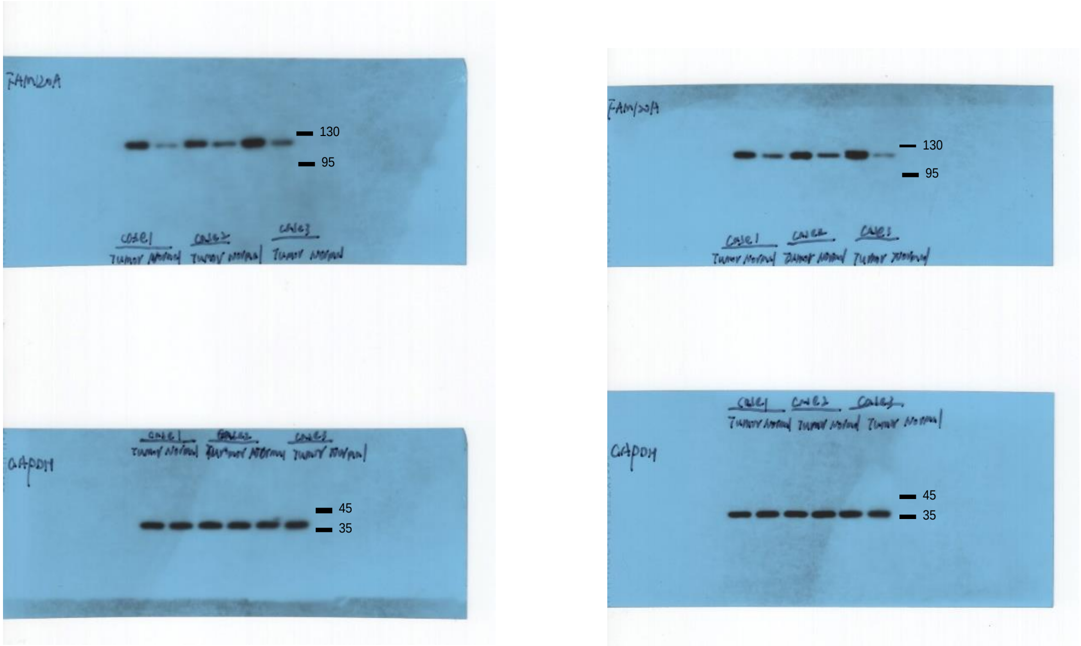

related to Figure 2i

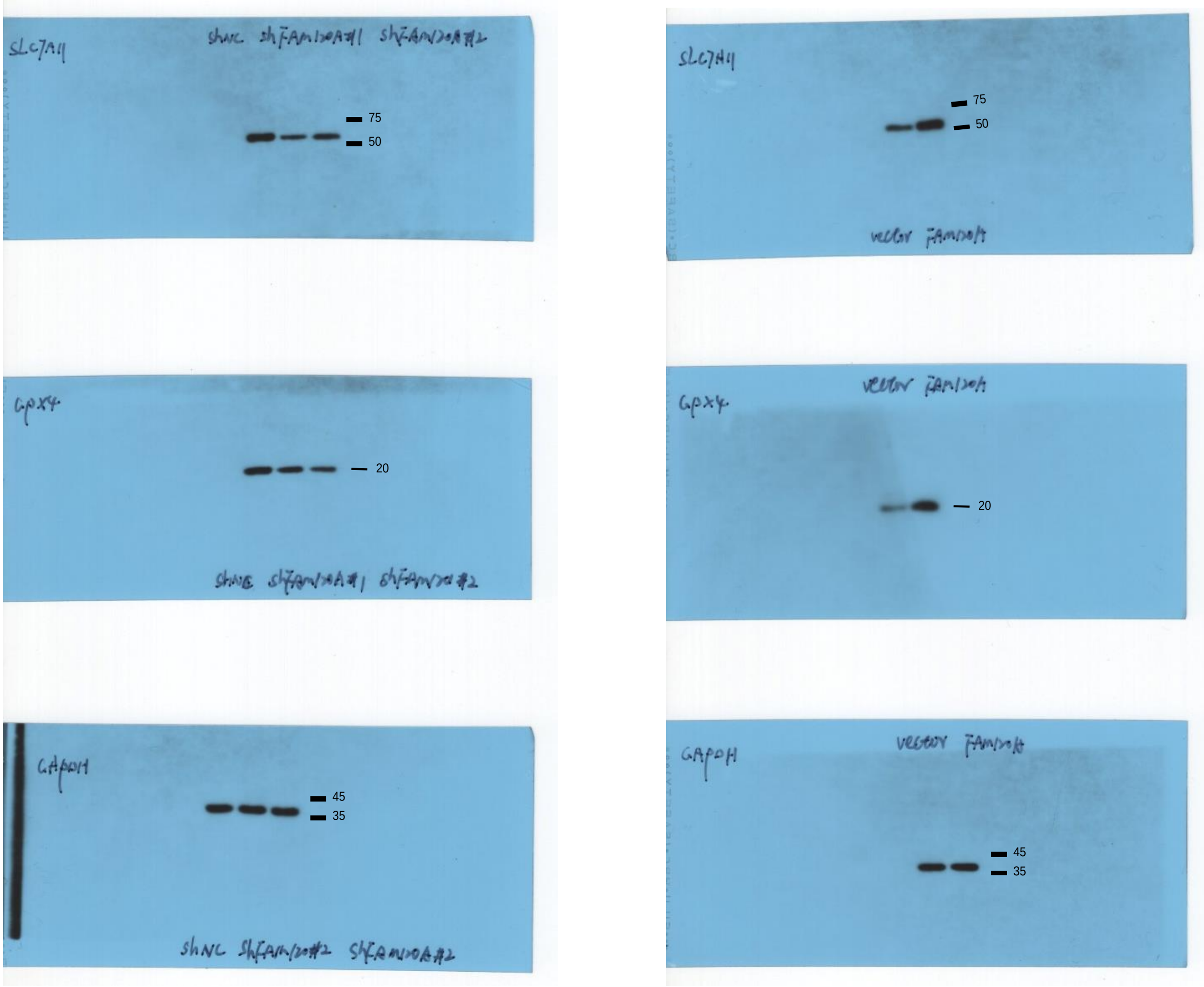

related to Figure 3l-m

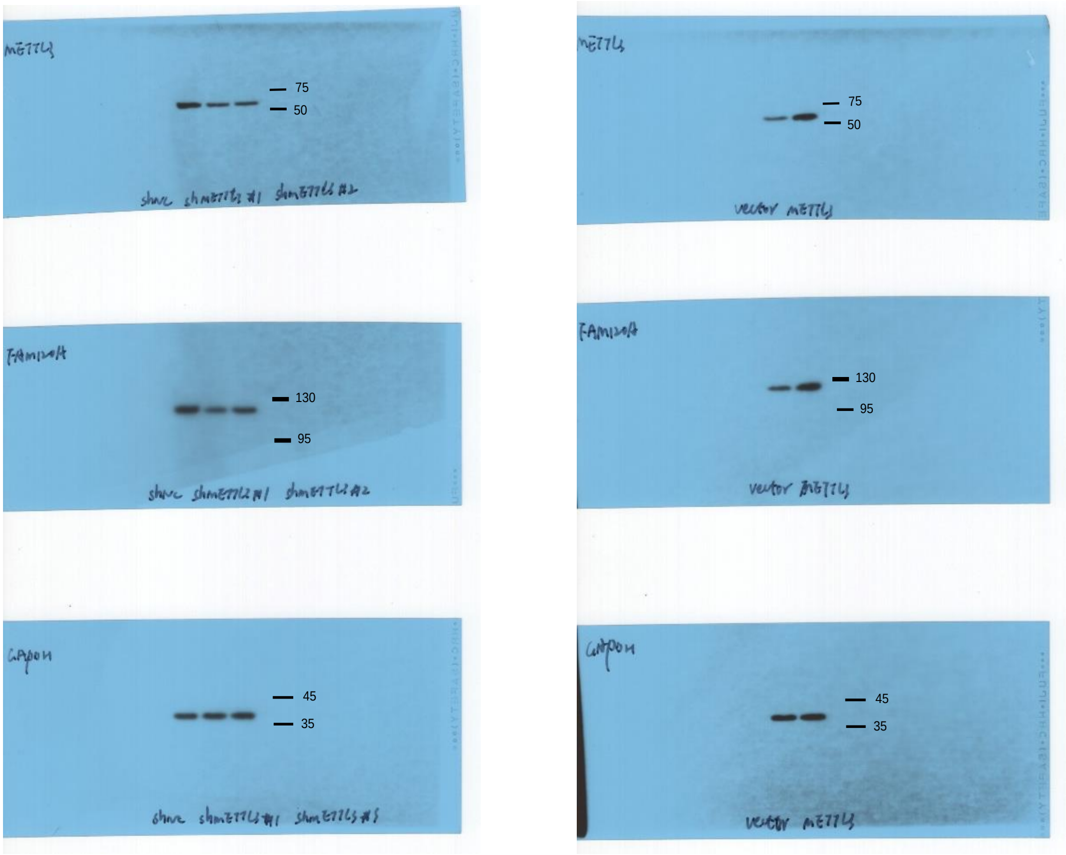

related to Figure 4g & 4k

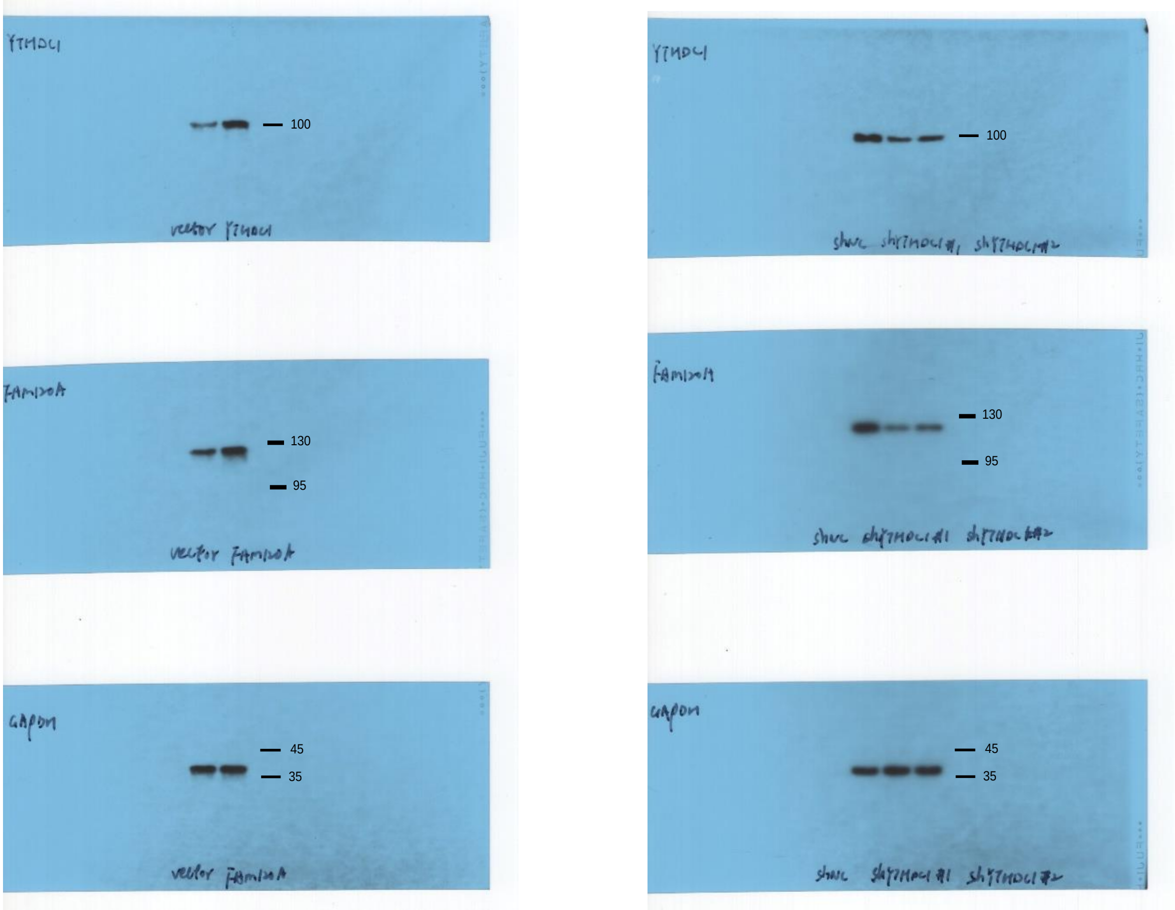

related to Figure 6g

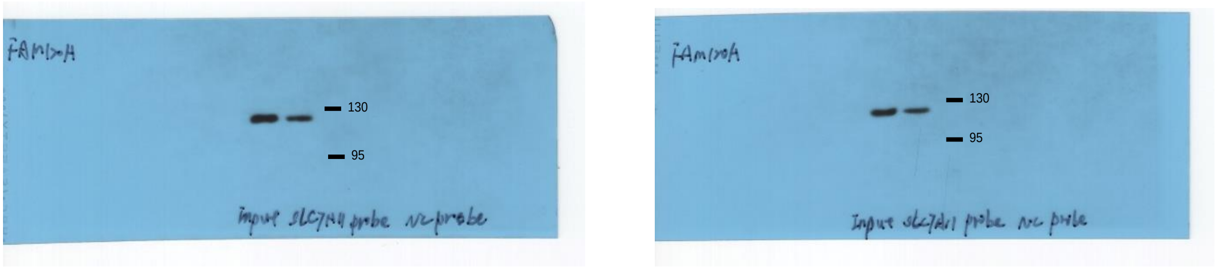

related to Figure 6j & 6n

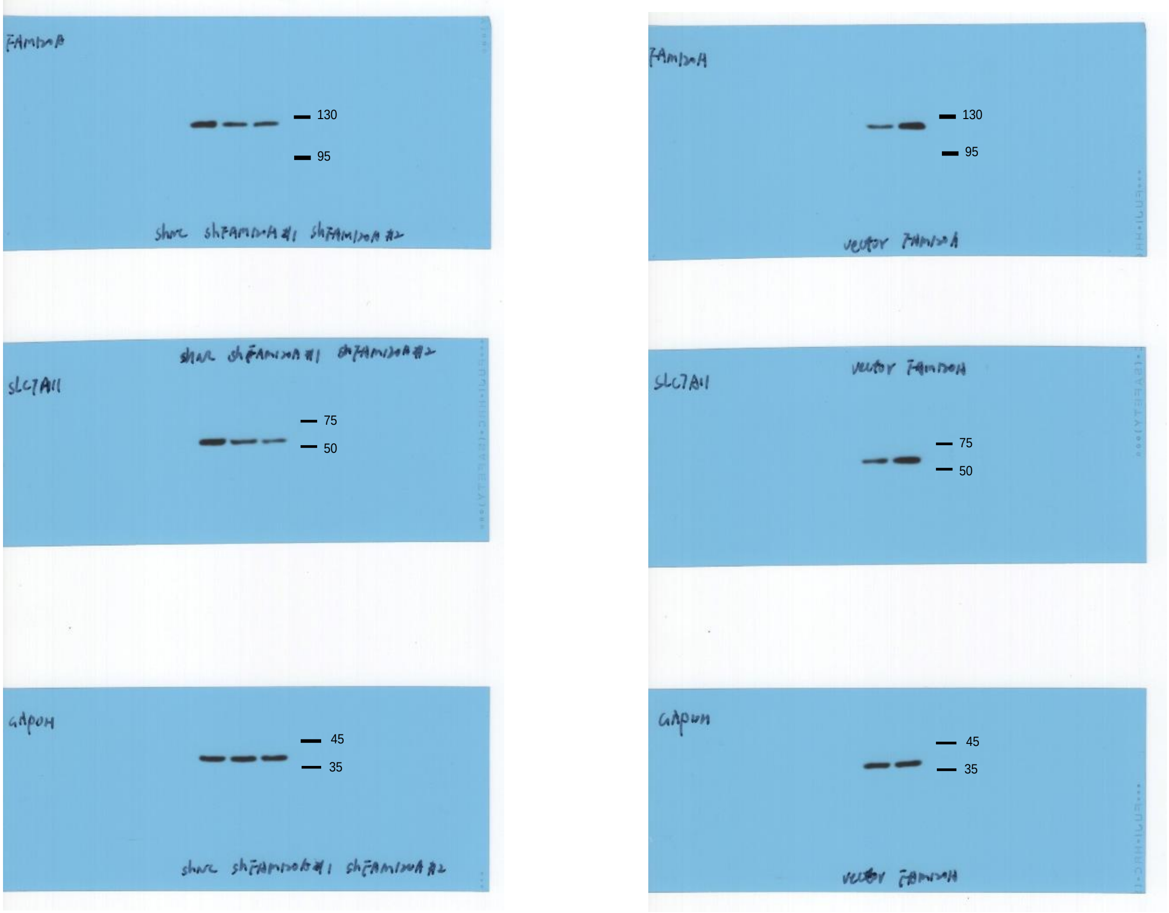

related to Figure S1a

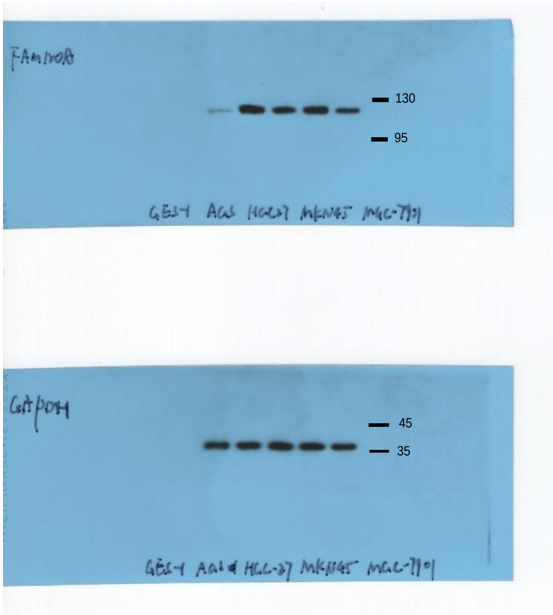

related to Figure S1d

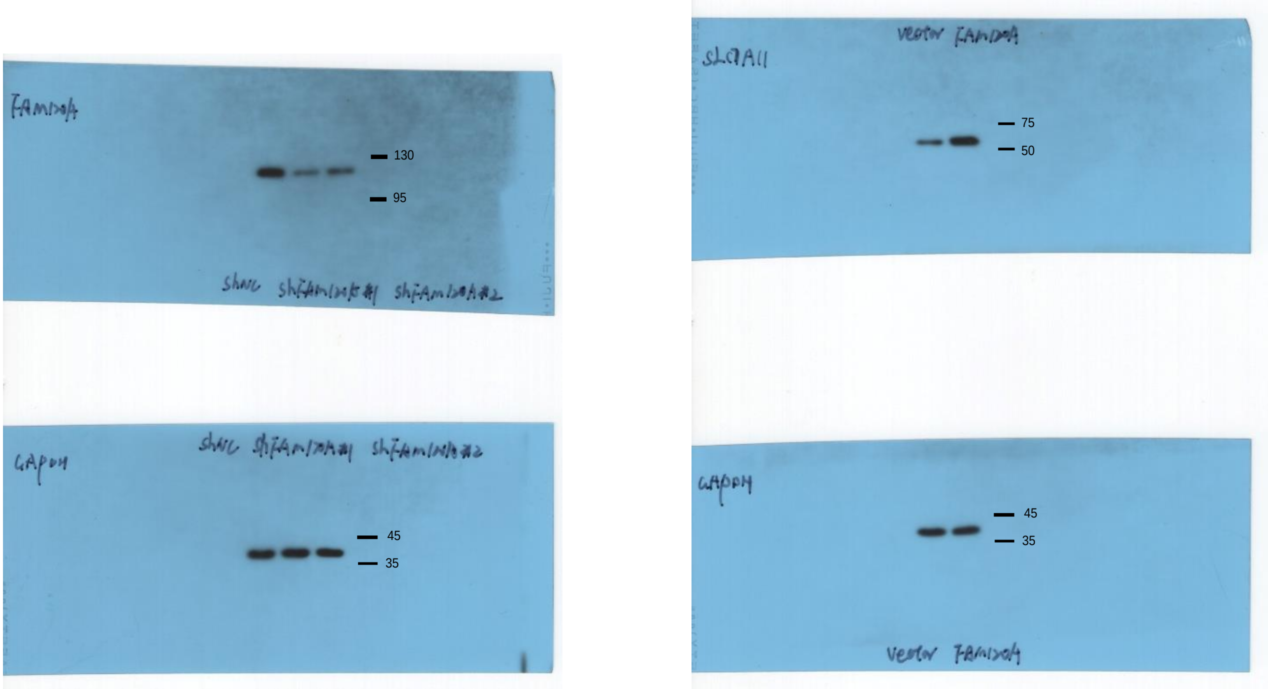

related to Figure S3d &3k

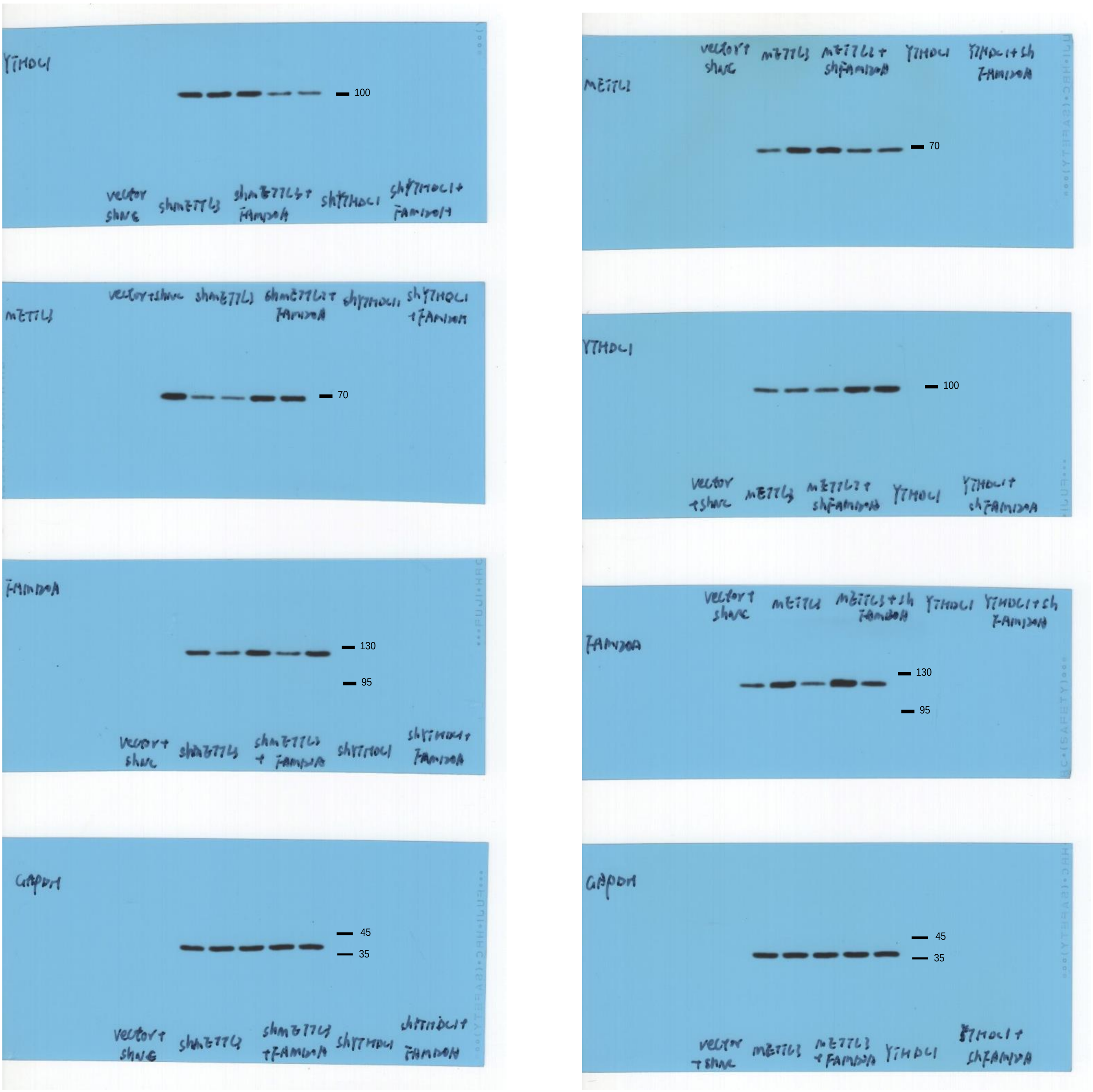

related to Figure S5b & 5h

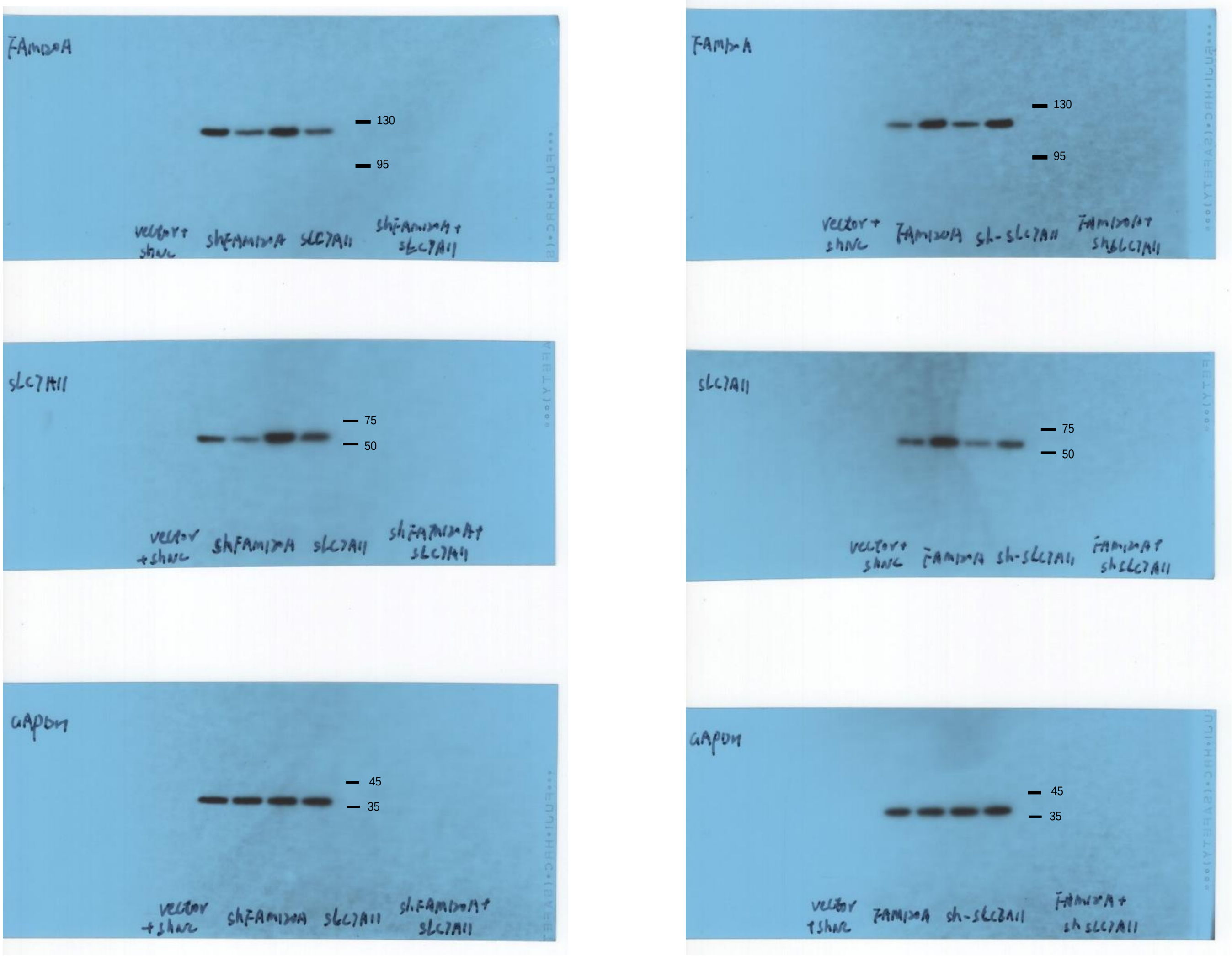

Supplement: Supplementary file 1 — Supplementary Information [file 42003_2024_6097_MOESM1_ESM.pdf]
