# Supplementary material for: FAM120A deficiency improves resistance to cisplatin in gastric cancer by promoting ferroptosis
Source: Commun Biol. 2024 Apr 2;7:399. doi: 10.1038/s42003-024-06097-6 (PMC10987584; doi:10.1038/s42003-024-06097-6)
Supplement: Supplementary file 2 — Description of Additional Supplementary Files [file 42003_2024_6097_MOESM2_ESM.pdf]

Description of Additional Supplementary Files

File name: Supplementary Data 1

Description: Source Data used to generate the main figures.
