# Supplementary material for: FAM120A deficiency improves resistance to cisplatin in gastric cancer by promoting ferroptosis
Source: Commun Biol. 2024 Apr 2;7:399. doi: 10.1038/s42003-024-06097-6 (PMC10987584; doi:10.1038/s42003-024-06097-6)
Supplement: Supplementary file 4 — Reporting summary [file 42003_2024_6097_MOESM4_ESM.pdf]

nature portfolio

Corresponding author(s): Wei HuangJ, ing Fu, Lu Feng

Last updated by author(s): Feb 28, 2024

Reporting Summary

Nature Portfolio wishes to improve the reproducibility of the work that we publish. This form provides structure for consistency and transparency in reporting. For further information on Nature Portfolio policies, see our Editorial Policies and the Editorial Policy Checklist.

Statistics

For all statistical analyses, confirm that the following items are present in the figure legend, table legend, main text, or Methods section.

n/a	Confirmed	
☐	☒	The exact sample size (*n*) for each experimental group/condition, given as a discrete number and unit of measurement
☐	☒	A statement on whether measurements were taken from distinct samples or whether the same sample was measured repeatedly
☐	☒	The statistical test(s) used AND whether they are one- or two-sided<br>*Only common tests should be described solely by name; describe more complex techniques in the Methods section.*
☒	☐	A description of all covariates tested
☐	☒	A description of any assumptions or corrections, such as tests of normality and adjustment for multiple comparisons
☐	☒	A full description of the statistical parameters including central tendency (e.g. means) or other basic estimates (e.g. regression coefficient) AND variation (e.g. standard deviation) or associated estimates of uncertainty (e.g. confidence intervals)
☐	☒	For null hypothesis testing, the test statistic (e.g. *F*, *t*, *r*) with confidence intervals, effect sizes, degrees of freedom and *P* value noted<br>*Give P values as exact values whenever suitable.*
☒	☐	For Bayesian analysis, information on the choice of priors and Markov chain Monte Carlo settings
☒	☐	For hierarchical and complex designs, identification of the appropriate level for tests and full reporting of outcomes
☐	☒	Estimates of effect sizes (e.g. Cohen's *d*, Pearson's *r*), indicating how they were calculated

Our web collection on statistics for biologists contains articles on many of the points above.

Software and code

Policy information about availability of computer code

Data collection	NA
Data analysis	NA

For manuscripts utilizing custom algorithms or software that are central to the research but not yet described in published literature, software must be made available to editors and reviewers. We strongly encourage code deposition in a community repository (e.g. GitHub). See the Nature Portfolio guidelines for submitting code & software for further information.

Data

Policy information about availability of data

All manuscripts must include a data availability statement. This statement should provide the following information, where applicable:

- Accession codes, unique identifiers, or web links for publicly available datasets
- A description of any restrictions on data availability
- For clinical datasets or third party data, please ensure that the statement adheres to our policy

The authers declare that the data supporting the findings of this study are available within the pare and its supplementary information files. The source data underlying the graphs in the paper can be found in the Supplementary Data 1. The Uncropped blots were shown in Supplementary Fig. S6.

Research involving human participants, their data, or biological material

Policy information about studies with human participants or human data. See also policy information about sex, gender (identity/presentation), and sexual orientation and race, ethnicity and racism.

Reporting on sex and gender	57 Male and 30 Female were included in this study.
Reporting on race, ethnicity, or other socially relevant groupings	All the test subjects were Asian.
Population characteristics	See above
Recruitment	The patients received operative treatment in the Sichuan Provincial People's Hospital between 2009 and 2017.
Ethics oversight	Written informed consent was acquired from all patients who participated in the study. The Ethics Committee of Sichuan Provincial People's Hospital approved the study.

Note that full information on the approval of the study protocol must also be provided in the manuscript.

Field-specific reporting

Please select the one below that is the best fit for your research. If you are not sure, read the appropriate sections before making your selection.

☒ Life sciences ☐ Behavioural & social sciences ☐ Ecological, evolutionary & environmental sciences

For a reference copy of the document with all sections, see nature.com/documents/nr-reporting-summary-flat.pdf

Life sciences study design

All studies must disclose on these points even when the disclosure is negative.

Sample size	A total of 87 GC tissues and their matched normal tissues were collected from patients who did not receive radiotherapy or chemotherapy enrolled in the Sichuan Provincial People's Hospital between 2009 and 2017.
Data exclusions	NA
Replication	The experiment was repeated three times independently
Randomization	NA
Blinding	The tester did not know the experimental grouping

Reporting for specific materials, systems and methods

We require information from authors about some types of materials, experimental systems and methods used in many studies. Here, indicate whether each material, system or method listed is relevant to your study. If you are not sure if a list item applies to your research, read the appropriate section before selecting a response.

Materials & experimental systems

n/a	Involved in the study	
☐	☒	Antibodies
☐	☒	Eukaryotic cell lines
☒	☐	Palaeontology and archaeology
☐	☒	Animals and other organisms
☐	☒	Clinical data
☒	☐	Dual use research of concern
☒	☐	Plants

Methods

n/a	Involved in the study	
☒	☐	ChIP-seq
☒	☐	Flow cytometry
☒	☐	MRI-based neuroimaging

Antibodies

Antibodies used	anti-FAM120A (1:200; Abcam, ab229254), anti-4-hydroxynonenal (4-HNE) (1:100; Abcam, ab48506), anti-Ki67 (1:200; Abcam, ab16667), anti-CD8 (1:2000; Abcam, ab217344). anti-N6-methyladenosine (m6A) (ab286164, Abcam), anti-METTL3 (ab195352, Abcam), Anti-YTHDC1 (ab220159, Abcam), Anti-FAM120A (ab156695, Abcam), FAM120A (Abcam, ab156695), GAPDH (CST, #2118),

	SLC7A11 (Abcam, ab307601), GPX4 (Abcam, ab41787), PD-L1 (CST, #13684), Anti-rabbit IgG (CST, #7074), Anti-mouse IgG (CST, #7076)
Validation	All antibodies are commercial antibodies purchased from Abcam and CST.

Eukaryotic cell lines

Policy information about cell lines and Sex and Gender in Research

Cell line source(s)	The normal gastric epithelial cell line GES-1 and GC cell lines (AGS, HGC27, MKN45, and SGC7901) were purchased from the Cell Bank of the Chinese Academy of Science (Shanghai, China)
Authentication	The normal gastric epithelial cell line GES-1 and GC cell lines (AGS, HGC27, MKN45, and SGC7901) were purchased from the Cell Bank of the Chinese Academy of Science (Shanghai, China)
Mycoplasma contamination	All cell lines tested negative for mycoplasma contamination
Commonly misidentified lines (See ICLAC register)	No

Animals and other research organisms

Policy information about studies involving animals; ARRIVE guidelines recommended for reporting animal research, and Sex and Gender in Research

Laboratory animals	M-NSG (NOD-PrkdcscidIl2rgem1/Smoc) mice
Wild animals	M-NSG (NOD-PrkdcscidIl2rgem1/Smoc) mice (5-6 weeks old) were obtained from the Shanghai Model Organisms Center Inc. (NM-NSG-001)
Reporting on sex	Female
Field-collected samples	Xenografts were collected
Ethics oversight	Experiments in mice were approved by the Laboratory Animal Ethics Committee of University of Electronic Science and Technology of China.

Note that full information on the approval of the study protocol must also be provided in the manuscript.

Clinical data

Policy information about clinical studies

All manuscripts should comply with the ICMJE guidelines for publication of clinical research and a completed CONSORT checklist must be included with all submissions.

Clinical trial registration	NA
Study protocol	Written informed consent was acquired from all patients who participated in the study. The Ethics Committee of Sichuan Provincial People's Hospital approved the study.
Data collection	Relevant clinical information was collected and sorted out during patient review and callback
Outcomes	NA
